# Efficacy of hematopoietic stem cell mobilization regimens in patients with hematological malignancies: a systematic review and network meta-analysis of randomized controlled trials

**DOI:** 10.1186/s13287-022-02802-6

**Published:** 2022-03-22

**Authors:** Chengxin Luo, Guixian Wu, Xiangtao Huang, Yali Zhang, Yanni Ma, Yarui Huang, Zhen Huang, Hui Li, Yu Hou, Jieping Chen, Xi Li, Shuangnian Xu

**Affiliations:** 1 Center for Hematology, Southwest Hospital, Third Military Medical University, #30 Gaotanyan Street, Shapingba District, Chongqing, 400038 China; 2 Key Laboratory of Cancer Immunotherapy of Chongqing, Chongqing, China; 3 Institute of Infectious Disease, Southwest Hospital, Third Military Medical University, #30 Gaotanyan Street, Shapingba District, Chongqing, 400038 China

**Keywords:** Hematopoietic stem cell mobilization, Hematological malignancies, G-CSF, Plerixafor, Cyclophosphamide

## Abstract

**Background:**

Efficient mobilization of hematopoietic stem cells (HSCs) from bone marrow niche into circulation is the key to successful collection and transplantation in patients with hematological malignancies. The efficacy of various HSCs mobilization regimens has been widely investigated, but the results are inconsistent.

**Methods:**

We performed comprehensive databases searching for eligible randomized controlled trials (RCTs) that comparing the efficacy of HSCs mobilization regimens in patients with hematological malignancies. Bayesian network meta-analyses were performed with WinBUGS. Standard dose of granulocyte colony-stimulating factor (G-CSF SD) was chosen as the common comparator. Estimates of relative treatment effects for other regimens were reported as mean differences (MD) or odds ratio (OR) with associated 95% credibility interval (95% CrI). The surface under the cumulative ranking curve (SUCRA) were obtained to present rank probabilities of all included regimens.

**Results:**

Databases searching and study selection identified 44 eligible RCTs, of which the mobilization results are summarized. Then we compared the efficacy of mobilization regimens separately for patients with multiple myeloma (MM) and non-Hodgkin lymphoma (NHL) by including 13 eligible trials for network meta-analysis, involving 638 patients with MM and 592 patients with NHL. For patients with MM, data are pooled from 8 trials for 6 regimens, including G-CSF in standard dose (SD) or reduced dose (RD) combined with cyclophosphamide (CY), intermediate-dose cytarabine (ID-AraC) or plerixafor. The results show that compared with G-CSF SD alone, 3 regimens including ID-AraC + G-CSF SD (MD 14.29, 95% CrI 9.99–18.53; SUCRA 1.00), G-CSF SD + Plerixafor SD (MD 4.15, 95% CrI 2.92–5.39; SUCRA 0.80), and CY + G-CSF RD (MD 1.18, 95% CrI 0.29–2.07; SUCRA 0.60) are associated with significantly increased total number of collected CD34^+^ cells (× 10^6^/kg), among which ID-AraC + G-CSF SD ranked first with a probability of being best regimen of 100%. Moreover, ID-AraC + G-CSF SD and G-CSF SD + Plerixafor SD are associated with significantly higher successful rate of achieving optimal target (collecting ≥ 4–6 × 10^6^ CD34^+^ cells/kg). For patients with NHL, data are pooled from 5 trials for 4 regimens, the results show that compared with G-CSF SD alone, G-CSF SD + Plerixafor SD (MD 3.62, 95% CrI 2.86–4.38; SUCRA 0.81) and G-CSF SD plus the new CXC chemokine receptor-4 (CXCR-4) antagonist YF-H-2015005 (MD 3.43, 95% CrI 2.51–4.35; SUCRA 0.69) are associated with significantly higher number of total CD34^+^ cells collected. These 2 regimens are also associated with significantly higher successful rate of achieving optimal target. There are no significant differences in rate of achieving optimal target between G-CSF SD + Plerixafor SD and G-CSF + YF-H-2015005.

**Conclusions:**

In conclusion, ID-AraC plus G-CSF is associated with the highest probability of being best mobilization regimen in patients with MM. For patients with NHL, G-CSF in combination with plerixafor or YF-H-2015005 showed similar improvements in HSCs mobilization efficacy. The relative effects of other chemotherapy-based mobilization regimens still require to be determined with further investigations.

**Supplementary Information:**

The online version contains supplementary material available at 10.1186/s13287-022-02802-6.

## Background

High-dose chemotherapy followed by autologous hematopoietic stem cell transplantation (HDT/ASCT) is a crucial therapeutic strategy for patients with hematological malignancies. For patients with newly diagnosed multiple myeloma (MM), high-risk or relapsed non-Hodgkin lymphoma (NHL) and Hodgkin lymphoma (HL), HDT/ASCT is part of standard care that could significantly prolonged progression-free survival and overall survival [1–6]. For leukemia patients who are ineligible for allogenic stem cell transplantation, consolidation therapy with HDT/ASCT decreased the risk of relapse and improved survival outcomes [7, 8]. In addition, ASCT provides a safer treatment platform with minimal treatment-related mortality compared with allogeneic stem cell transplantation [2, 9]. Over the past decades, peripheral blood stem cells (PBSCs) have largely replaced bone marrow as the predominant source of stem cells for autologous transplantation due to the convenient collection procedure and rapid hematologic recovery [10, 11]. The collection of sufficient high-quality autologous PBSCs relies on the successful mobilization of hematopoietic stem cells (HSCs) from bone marrow niche into circulation. To ensure successful multi-lineage engraftment after transplantation, a minimal dose of 2 × 10^6^ CD34^+^ cells per kilogram (kg) body weight and an optimal dose is 5 × 10^6^ CD34^+^ cells/kg are required [12, 13]. Increase in the doses of reinfused stem cells leads to better post-transplantation clinical outcomes [14]. Therefore, successful HSCs mobilization is a crucial part of efficient treatment in patients with hematological malignancies.

Granulocyte colony-stimulating factor (G-CSF) is the most commonly used mobilization agent in clinical practice. G-CSF alone can induce effective HSCs mobilization through complicated mechanisms such as triggering the release of proteolytic enzymes and disrupting the stromal derived factor-1 (SDF-1)/CXC chemokine receptor-4 (CXCR-4) axis [12, 15]. However, patients with hematological malignancies are associated with increased risk of mobilization failure due to the poor bone marrow reserve resulted from repetitive exposure to the toxicity of chemotherapy and radiation therapy [16]. Mobilization with G-CSF alone fail to yield adequate CD34^+^ cells in approximately 5–30% of patients with MM or lymphoma [13, 14]. Therefore, the HSCs mobilization ability of other agents has been widely investigated, such as granulocyte–macrophage colony-stimulating factor (GM-CSF), stem cell factor (SCF), and the CXCR-4 antagonist plerixafor [17]. In addition, chemotherapeutic regimens, especially cyclophosphamide, are commonly used in combination with growth factors for autologous HSCs mobilization, which could improve CD34^+^ cells yield and reduce tumor cells burden, but with the expense of increased toxicity [18]. The efficacy and safety of various mobilization agents and regimens are compared with a series of high-quality randomized controlled trials (RCTs). However, the optimal mobilization approaches are still not well-established due to the inconsistency in results and the paucity of direct comparisons among several important mobilization strategies.

In this study, we aimed to perform a systematic review and network meta-analysis to compare the efficacy of mobilization regimens in patients with hematological malignancies, hoping to provide high-level evidence for decision making in clinical practice via synthesizing available direct and indirect evidence from relevant RCTs.

## Methods

### Literature search and study selection

This study is conducted in accordance with the Preferred Reporting Items for Systematic Reviews and Meta-analyses (PRISMA) extension statement for network meta-analyses [19]. We searched Medline (by Ovid), Embase, Cochrane library and China Biology Medicine (CBM) databases from inception to April 22, 2021 without language restrictions for randomized controlled trials (RCTs) that comparing the efficacy of HSCs mobilization regimens in patients with hematological malignancies. The search terms including MeSH term ‘hematopoietic stem cell mobilization’, free text ‘stem cell mobilization’ and ‘randomized controlled trial’. Reference lists of included trials and relevant reviews were manually searched for additional trials.

Two investigators (CXL and GXW) independently assessed the eligibility of retrieved citations. Disagreements were resolved by discussion with a third investigator (SNX). RCTs comparing the efficacy of two or more mobilization regimens in patients with hematological malignancies were included. The inclusion criteria were: (i) included patients with primary diagnosis of hematological malignancies and eligible for ASCT; (ii) randomly assigned patients to receive two or more kinds of HSCs mobilization regimens; (iii) reported data for at least one of the efficacy outcomes, including the total number of collected CD34^+^ cells per kilogram (kg) of body weight, and the proportions of patients achieving optimal mobilization target (collecting ≥ 4–6 × 10^6^ CD34^+^ cells/kg). We excluded quasi-randomized studies, dose-escalating studies, pharmacokinetic studies, cost-effectiveness analyses, post-hoc analyses, studies on healthy volunteers, and studies investigating the effects of mobilization agents on hematologic recovery. Studies including patients with malignancies of other systems (such as breast cancer, lung cancer, osteosarcoma, germ cell tumors and other solid tumors) were also excluded in the review. For network meta-analysis, studies that concurrently included patients with different hematological malignancies without providing subgroup results were excluded to reduce heterogeneity. Since the mobilization strategies and support therapies changed a lot over time, studies that enrolled participants before the year of 2000 were excluded.

### Data extraction and quality assessment

Two investigators (CXL and GXW) independently extracted data using predesigned data collection forms. Results are cross-checked to reach a consensus. The extracted data include trial characteristics, patient characteristics, dosage and duration of mobilization agents, and efficacy outcomes. The primary outcome is total number of collected CD34^+^ cells (× 10^6^/kg), secondary outcome is the successful mobilization rate (described as the proportions of patients collecting ≥ 4–6 × 10^6^ CD34^+^ cells/kg). For continuous outcomes, the mean value and standard deviation were directly extracted, or estimated from median, range and sample size using previously validated methods [20]. For trials with multiple publications, we included all reports and extracted data from the most informative and complete one. Risk of bias for each included trial was assessed based on random sequence generation, allocation concealment, blinding, incomplete outcome data and selective outcome reporting, following the guidelines in Cochrane handbook [21]. Any disagreements were resolved by consensus.

### Statistical analyses

We conducted Bayesian network meta-analyses following the guidelines of the National Institute for Health and Care Excellence Decision Support Unit (NICE DSU) [22]. Network meta-analyses were performed with WinBUGS version 1.4.3 (MRC Biostatistics Unit, Cambridge, UK), employing the Markov Chain Monte Carlo (MCMC) approach. We used the WinBUGS code previously established by Dias et al*.*, which could handle trials with multiple arms and rank treatments with additional code [22]. Three chains were run to yield 150,000 iterations, and the initial 5000 burn-ins were discarded. The convergence of model was assessed with trace plots and Brooks-Gelman-Rubin statistic. Model fit of fixed-effect model and random-effect model were compared with the Deviance Information Criterion (DIC), and model with lower DIC was adopted. Standard dose of G-CSF (G-CSF SD) was chosen as the common comparator. Estimates of relative treatment effects were reported as mean differences (MD) or odds ratio (OR) with the associated 95% credibility interval (95% CrI). The 95% CrI calculated in Bayesian meta-analysis can be interpreted like the 95% confidence intervals (95% CI) in traditional meta-analysis. Extra codes are used to obtain rank results, the probability of being best regimen and the surface under the cumulative ranking curve (SUCRA) for each regimen [22, 23]. The publication bias was assessed with comparison-adjusted funnel plot. We used Stata version 13.0 to create the network plots, comparison-adjusted funnel plot and SUCRA plots.

## Results

### Characteristics of included trials

**Fig. 1:**
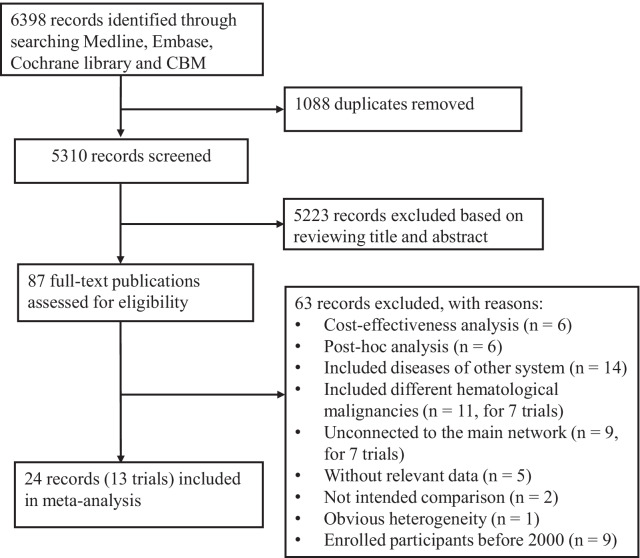
PRISMA flowchart of study selection

Databases searching identified 6398 potentially relevant references, of which 1088 duplicates were removed and 5223 records were excluded based on reviewing title and abstract. Full-text publications of the remaining 87 records were retrieved for further evaluation. After excluded studies of cost-effectiveness analysis and post-hoc analysis, and studies that included other diseases, 44 trials were included for systematic review [24–67]. For further evaluation, different doses of G-CSF or Biosimilar G-CSF are classified into 2 groups as previously described: standard dose (SD, 10 μg/kg/day or 400 μg/m^2^/day), reduced dose (RD, 5–7.5 μg/kg/day or 250 μg/m^2^/day) [57, 68]. Different doses of plerixafor are classified as SD (standard dose, 0.24 mg/kg/day) and FD (fixed dose, 20 mg/day). Two trials comparing different administration schedules of a same regimen (single versus split dose, early versus late administration) were excluded for final analysis since they are not intended comparison [41, 53]. To reduce heterogeneity, one trial with obvious differences in mobilization target and maximum apheresis was excluded [29]. In addition, we excluded 7 trials that concurrently included different hematological malignancies without providing subgroup results, 9 trials that unconnectd to the network, 5 trials that did not have relevant data of the mobilization outcome, and 9 trials that enrolled participants before the year of 2000. Ultimately, 13 eligible trials were included for the network meta-analysis, including 8 trials for MM and 5 trials for NHL. The flow chart depicting study selection is shown in Fig. 1.

The characteristics of the 44 trials included in review are summarized in Table 1. In brief, Mobilization regimens investigated include G-CSF alone, mobilizing chemotherapy in combination with G-CSF or/and other cytokines, and G-CSF in combination with CXCR4 antagonists (plerixafor or YF-H-2015005). Other cytokines include erythropoietin (EPO), interleukin 11 (IL-11), GM-CSF, SCF and thrombopoietin (TPO). As for mobilizing chemotherapy regimens, the most commonly used regimen is cyclophosphamide (CY) alone. The detailed information for all mobilizing chemotherapy regimens is provided in Additional file 1: Table S1. For patients with MM, intermediate-dose cytarabine (ID-AraC), gemcitabine and vinorelbine are also used for mobilizing chemotherapy. For patients with NHL, salvage therapy regimens such as DHAP (dexamethasone, high-dose cytarabine, and cisplatin), ESHAP (etoposide, methylprednisolone, high-dose cytarabine, and cisplatin), and ICE (ifosfamide, carboplatin and etoposide) are also used for mobilizing chemotherapy. A total of 49 mobilization regimens were investigated, the specific dosage of all mobilization regimens is provided in Additional file 2: Table S2.

**Table 1:** The characteristics and mobilization results of the 44 studies included in review

References	Study design	Enroll period	Patients	Mobilization regimen	Main results
**G-CSF plus Plerixafor SD versus G-CSF alone**					
DiPersio et al. [32]	Phase 3 RCT, double-blind, multicenter	Jan. 2005–Aug. 2006	NHL	G-CSF SD; G-CSF SD + Plerixafor SD	The Plerixafor group showed significantly higher rate of achieving optimal target (*P* < 0.001)* and higher CD34^+^ cells collected
DiPersio et al. [33]	Phase 3 RCT, double-blind, multicenter	Feb. 2005–Jul. 2006	MM	G-CSF SD; G-CSF SD + Plerixafor SD	The Plerixafor group showed significantly higher rate of achieving optimal target (*P* < 0.001)* and higher CD34^+^ cells collected
Matsue et al. [48]	Phase 2 RCT, open-label, multicenter	Nov. 2014–Mar. 2016	NHL	G-CSF SD; G-CSF SD + Plerixafor SD	The Plerixafor group showed higher rate of achieving optimal target
Nahi et al. [50]	Phase 2 RCT, open-label, multicenter	NA	MM	G-CSF SD; G-CSF SD + Plerixafor SD	The Plerixafor group showed higher CD34^+^ cells yield
Ri et al. [55]	Phase 2 RCT, open-label, multicenter	Oct. 2014–Jul. 2015	MM	G-CSF SD; G-CSF SD + Plerixafor SD	The Plerixafor group showed higher rate of achieving optimal target
Zhu et al. [66]	Phase 3 RCT, double-blind, multicenter	Apr. 2013–Nov. 2014	NHL	G-CSF SD; G-CSF SD + Plerixafor SD	The Plerixafor group showed significantly higher rate of achieving optimal target (*P* < 0.0001)*
**G-CSF plus YF-H-2015005 versus G-CSF alone**					
Liu et al. [44]	Phase 3 RCT, double-blind, multicenter	Jan. 2017- Dec. 2018	NHL	G-CSF SD; G-CSF SD + YF-H-2015005	YF-H-2015005 significantly increased the rate of achieving optimal target (*P* < 0.001)* and the number of CD34^+^ cells collected (*P* < 0.001)*
**Chemotherapy regimens plus G-CSF versus G-CSF alone**					
Karanth et al. [40]	RCT, open-label, single center	Nov. 1998–Nov. 2002	HL, NHL, MM or CLL	G-CSF SD; CY + G-CSF RD	No significant differences in rate of successful mobilization (*P* = 0.21) and the number of CD34^+^ cells collected
Silvennoinen et al. [58]	Phase 2 RCT, multicenter	Jan. 2013- Feb. 2015	MM	G-CSF SD; CY + G-CSF RD	CY plus G-CSF RD resulted in significantly higher CD34^+^ cells yield (*P* = 0.012)*
Valtola et al. [61]	RCT, multicenter	Jan. 2013–Nov. 2014	MM	G-CSF SD; CY + G-CSF RD	No significant differences in the number of total CD34^+^ cells collected (*P* = 0.064)
Milone et al. [49]	RCT, single center	Mar. 1998–Jul. 2002	NHL or HL	G-CSF SD; CY + G-CSF SD	No significant differences in the number of CD34^+^ cells collected (*P* > 0.9) and the rate of successful mobilization (*P* > 0.4)
Narayanasami et al. [51]	RCT, single center	Nov. 1997–Nov. 2000	NHL or HL	G-CSF SD; CY + G-CSF SD	CY plus G-CSF SD resulted in significantly higher total number of CD34 + cells collected (*P* = 0.004*)
Czerw et al. [30]	Phase 3 RCT, open-label, single center	Mar. 2013–Mar. 2016	MM	G-CSF SD; ID-AraC + G-CSF SD	The ID-AraC group showed significantly higher total number of CD34^+^ cells collected (*P* < 0.000001) and higher rate of reaching optimal target (*P* = 0.0003)*
**Comprisons among chemotherapy regimens**					
Chen et al. [28]	RCT, single center	Jan. 2005–May 2010	NHL	CY + G-CSF RD; MA + G-CSF RD	The number of total CD34^+^ cells collected were similar (*P* = 0.117)
Pavone et al. [54]	RCT, single center	NA	NHL	CY + G-CSF RD; DHAP + G-CSF RD	No significant differences in the mean number of CD34^+^ cells collected
Vela-Ojeda et al. [62]	RCT, single center	Aug. 1994–Jun. 1999	NHL, HL or MM	CY + GM-CSF RD; Ifosfamide + GM-CSF RD	No significant differences in the number of CD34^+^ cells collected (*P* = 0.1)
Jeker et al. [38]	Phase 2 RCT, single center	Dec. 2013–Apr. 2017	MM	Vinorelbine + G-CSF SD; Gemcitabine + G-CSF SD	The CD34^+^ cell yield was significantly higher in the Vinorelbine group (*P* = 0.001)*
Weaver et al. [63]	RCT, single center	Sept. 1992–Aug. 1994	NHL or HL	CE + G-CSF RD; CEP + G-CSF RD	No significant difference in the number of total CD34^+^ cells collected (*P* = 0.09)
Zhang et al. [64]	RCT, single center	Jan. 2001–Oct. 2012	NHL	MEOD + G-CSF SD; MEOD + MTX + G-CSF SD	The MTX group showed significantly higher CD34^+^ cells yield (*P* < 0.05)*
**Pegfilgrastim versus G-CSF**					
Bouko et al. [26]	Phase 2 RCT	May 2006–Nov. 2011	MM	G-CSF SD; Pegfilgrastim 12 or 18 mg	No significant differences in the rate of reaching minimal and optimal target
Kuan et al. [42]	RCT, triple blinded, single center	Sep. 2010–Dec. 2012	Acute leukemia, MM or lymphoma	CY + G-CSF RD; CY + Pegfilgrastim 6 mg on day 3 or day 7	Pegfilgrastim 6 mg on day 7 produced highest rate of successful mobilization
Russell et al. [56]	Phase 2 RCT, double-blind, multicenter	Feb. 2003–Sep. 2004	NHL	ICE + G-CSF RD; ICE + Pegfilgrastim 6 mg or 12 mg	No significant differences in the number of CD34^+^ cells collected and the rate of reaching optimal target
Skopec et al. [59]	RCT, single center	Feb. 2012–Nov. 2014	MM	G-CSF SD; Pegfilgrastim 12 mg	No significant difference in the number of CD34^+^ cells collected (*P* = 0.428)
**Biosimilar G-CSF versus G-CSF**					
Bhamidipati et al. [25]	Phase 2 RCT, open-label, single center	Aug. 2014–Jun. 2016	MM or NHL	G-CSF SD + Plerixafor SD; Biosimilar G-CSF (Tbo-filgrastim) + Plerixafor SD	No significant differences in the number of CD34^+^ cells collected on day 5 (*P* = 0.873) and successful rate of reaching optimal target (*P* = 0.916)
Manko et al. [46]	RCT, single center	Jun. 2010–Sep. 2011	MM, NHL, HL, AML or other	Chemotherapy + G-CSF SD; Chemotherapy + Biosimilar G-CSF SD;	No significant differences in the number of CD34 + cells collected and the rate of successful mobilization
Marchesi et al. [47]	RCT, single center	Oct. 2014–Nov. 2017	NHL or HL	Chemotherapy + G-CSF RD; Chemotherapy + Biosimilar G-CSF SD	No significant differences in the number of CD34^+^ cells collected (*P* = 0.805) and the rate of achieving optimal target (*P* = 1.00)
**GM-CSF versus G-CSF**					
Arora et al. [24]	RCT, single center	1993–2002	MM	CMD + G-CSF RD; CMD + GM-CSF RD	Two group showed similar CD34^+^ cells collection (*P* = 0.8). G-CSF is associated with faster neutrophil and platelet recovery
Demuynck et al. [31]	RCT, single center	NA	MM	CY + G-CSF SD; CY + GM-CSF SD	No significant difference in CD34^+^ cells yield (*P* = 0.27). GM-CSF is associated with increased toxicity
Gazitt et al. [35]	RCT, single center	May 1997–Mar. 2000	NHL	CY + G-CSF SD; CY + GM-CSF RD; CY + GM-CSF RD + G-CSF SD	No significant differences in successful rate of collecting ≥ 2 × 10^6^ CD34^+^CD45^dim^ cells/kg
Hohaus et al. [37]	RCT, double-blind, single center	Aug. 1992–Dec. 1994	HL	G-CSF RD; GM-CSF RD	No significant differences in the number of CD34^+^ cells collected (*P* = 0.696)
**SCF versus no SCF**					
Bourin et al. [27]	RCT, single center	NA	MM	CY + G-CSF RD; SCF + G-CSF SD	The total number of CD34^+^ cells collected were similar
Facon et al. [34]	RCT, open-label, multicenter	Mar. 1996–Oct. 1997	MM	CY + G-CSF RD; CY + SCF + G-CSF RD	The SCF group showed significant higher CD34^+^ cells yield (*P* = 0.007)*
Johnsen et al. [39]	Phase 2 RCT, open-label, multicenter	NA	Malignant lymphoma	CY + G-CSF SD; SCF + G-CSF SD	The CY plus G-CSF group showed higher number of CD34^+^ cells collected and higher rate of reaching optimal target on first leukapheresis (*P* = 0.00018)*
Stiff et al. [60]	RCT, multicenter	NA	NHL or HL	G-CSF SD; SCF + G-CSF SD	SCF group showed an increase in the median total CD34^+^ cells collected (*P* = 0.05)
**Addition of other cytokines**					
Hart et al. [36]	RCT, single center	May 2004–Jan 2006	MM	IEV + G-CSF RD; IEV + G-CSF RD + EPO	No significant differences in the number of CD34^+^ cells collected (*P* = 0.57)
Lonial et al. [45]	RCT, single center	NA	Lymphoma or MM	Chemotherapy + G-CSF SD; Chemotherapy + G-CSF RD + GM-CSF RD	No significant differences in the number of CD34^+^ cells collected
Zhu et al. [65]	RCT, single center	2002–2005	NHL or AML	Chemotherapy + G-CSF RD; Chemotherapy + G-CSF RD + IL-11	No significant differences in the number of total CD34^+^ cells collected
Zhu et al. [67]	RCT, multicenter	NA	NHL	CE + G-CSF RD; CE + G-CSF RD + TPO	The TPO group showed significantly higher total number of CD34^+^ cells collected (*P* = 0.0054) and higher rate of reaching optimal target (*P* = 0.021)*
**Comparison of different administration schedules**					
Kim et al. [41]	RCT, single center	Jun. 2003–Oct. 2004	MM or NHL	CY or ESHAP (± Rituximab) + G-CSF SD (single-dose versus split-dose)	No significant differences in the number of CD34^+^ cells collected (*P* = 0.47) and rate of reaching optimal target (*P* = 0.24)
Kuruvilla et al. [43]	Phase 4 RCT, open-label, multicenter	Oct. 2010–Feb. 2013	NHL	G-CSF SD + Plerixafor SD; G-CSF SD + Plerixafor FD	No significant differences in the rate of achieving optimal target (*P* = 0.395)
Ozcelik et al. [53]	RCT, single center	2005–2008	NHL or MM	CE + G-CSF SD (early versus late)	No significant differences in the number of CD34^+^ cells collected (*P* = 0.781)
Samaras et al. [57]	Phase 2 RCT, single center	2011–2016	MM	Vinorelbine + G-CSF SD; Vinorelbine + G-CSF RD	No significant differences in the number of CD34^+^ cells collected (*P* = 0.99)
**Others**					
Copelan et al. [29]	RCT, single center	May. 2000–Apr. 2005	B-cell NHL	VP-16 + G-CSF SD; Rituximab + VP-16 + G-CSF SD	The Rituximab group showed significantly higher total number of CD34 + cells collected (*P* = 0.021)*
Orciuolo et al. [52]	RCT, open-label, multicenter	Apr. 2005–Jul. 2009	MM	CY + G-CSF SD (lenograstim versus filgrastim)	No significant differences in the rate of reaching optimal target

AML, acute myelocytic leukemia; CE, cyclophosphamide and etoposide; CEP, cyclophosphamide and etoposide plus cisplatin; CLL, chronic lymphocytic leukemia; CMD, cyclophosphamide, mitoxantrone and dexamethasone; CY, cyclophosphamide; DHAP, dexamethasone, high-dose cytarabine, and cisplatin; ESHAP, etoposide, methylprednisolone, high-dose cytarabine, and cisplatin; EPO, erythropoietin; FD, fixed dose; GM-CSF, granulocyte–macrophage colony-stimulating factor; G-CSF: granulocyte colony-stimulating factor; HL, Hodgkin lymphoma; ICE, ifosfamide, carboplatin and etoposide; ID-AraC, intermediate-dose cytarabine; IEV, ifosfamide, epirubicin and etoposide; IL-11, interleukin 11; MA, methotrexate, cytarabine; MEOD, mitoxantrone, etoposide, vindesine and dexamethasone; MM, multiple myeloma; MTX, methotrexate; NA, not available; NHL, non-Hodgkin lymphoma; RCT, randomized controlled trial; RD, reduced dose; SCF, stem cell factor; SD, standard dose; TPO, thrombopoietin; VP-16, etoposide; YF-H-2015005, a new CXCR4 antagonist

*Results with significant difference

After the administration of mobilization regimens, apheresis procedure was initiated on day 5 in patients mobilized with G-CSF alone or G-CSF plus CXCR4 antagonists. In patients mobilized with chemotherapy-based regimens, apheresis procedure was often initiated when peripheral blood (PB) white blood cells count recovery to more than 1 × 10^9^/L or when PB CD34^+^ cells > 10/μL, required a median interval from drug administration to apheresis initiation of 7–15 days. The end point of apheresis is the achievement of minimal collection target (≥ 2 × 10^6^ CD34^+^ cells/kg) or optimal collection target (≥ 4–6 × 10^6^ CD34^+^ cells/kg). The allowed maximum number of apheresis ranges from 3 to 5. The mobilization target and allowed maximum number of apheresis for all studies included in review are listed in Additional file 3: Table S3.

The main mobilization results of the 44 studies included in review are summarized in Table 1. Briefly, G-CSF SD plus Plerixafor significantly increased the number of CD34^+^ cells collected and the successful rate of achieving optimal target in patients with MM and NHL. For patients with NHL, the addition of another CXCR4 antagonists YF-H-2015005 also significantly improved mobilization efficacy in comparison with G-CSF alone. As for CY plus G-CSF, the results of mobilization efficacy comparison versus G-CSF alone varies in included studies. Silvennoinen et al*.* reported that CY plus G-CSF RD significantly increased the number of harvested CD34^+^ cells versus G-CSF SD in patients with MM, and Narayanasami et al*.* reported that CY plus G-CSF SD significantly increased the number of harvested CD34^+^ cells versus G-CSF SD in patients with NHL and HL, but another 3 studies did not report any statistically significant difference [40, 49, 51, 58, 61]. As for other mobilizing chemotherapy regimens, ID-AraC plus G-CSF SD significantly impproved mobilization efficacy over G-CSF SD alone in patients with MM, RCT comparing the other chemotherapy regimens plus G-CSF with G-CSF alone is not available [30]. The mobilization efficacy of salvage therapy regimen DHAP is compared with that of CY in patients with NHL, the results suggested no significant difference in the mean number of CD34^+^ cells collected (5.9 versus 7.06) [54]. Comparisons among other mobilizing chemotherapy reimens suggested that vinorelbine showed superior efficacy than gemcitabine in MM patients, the addition of methotrexate (MTX) to MEOD (mitoxantrone, etoposide, vindesine and dexamethasone) plus G-CSF can improve mobilization efficacy in NHL patients [38, 64].

The mobilization efficacy of pegylated G-CSF (pegfilgrastim) and Biosimilar G-CSF are compared with G-CSF, and similar results in the number of CD34^+^ cells collected and the rare of achieving minimal/optimal target are reported (Table 1). As for other cytokines, GM-CSF is compared with G-CSF in several RCTs published between 1995 and 2004. GM-CSF showed no advantage in mobilization efficacy, but is associated with increased toxicity and later engrafment than G-CSF. Addition of SCF improved the mobilization efficacy of CY plus G-CSF RD in patients with MM, but SCF plus G-CSF is not superior to CY plus G-CSF [27, 34, 39, 60]. Addition of TPO to mobilizing chemotherapy plus G-CSF significantly increased the number of CD34^+^ cells collected and the rare of achieving optimal target in patients with NHL, but addition of other cytokines including EPO, GM-CSF and IL-11 did not show significant improvement [36, 45, 65, 67]. For patients with B-cell NHL, priming with rituximab improved mobilization efficacy significantly [29]. Other comparisons did not report significant differences in mobilization efficacy (Table 1). The number of CD34 + cells collected, proportions of patients reaching minimal and optimal target, and the time to neutrophil and platelet engraftment after ASCT for each study are provided in Additional file 3: Table S3.

The characteristics of the 13 trials that included in the network meta-analysis are presented in Table 2. Overall, 1230 patients with hematological malignancies were involved, including 638 patients with MM, and 592 patients with NHL. The results of quality assessment are shown in Additional file 4: Table S4. Random sequence generation are adequate in 8 trials, and allocation sequence concealment are adequate in 5 trials. The other trials did not provide sufficient information to evaluate selection bias. As for blinding of participants and personnel, only 4 trials reported a double-blind design, whereas 5 trials are open-label. As for blinding of outcome assessment, the risk of bias resulted from non-blind outcome assessment are low since the mobilization outcomes are all objective measurements. All of the included trials are free from attrition bias, reporting bias and any other bias. The comparison-adjusted funnel plot is shown in Additional file 6: Figure S1.

**Table 2:** Characteristics of the 17 trials included in meta-analysis

Study	Eligible patients	Mobilization regimen	No. of patients	Age*	Gender (male%)
**Studies for MM**					
Bouko et al. [26]	Newly diagnosed MM, responders to 3–4 cycles of induction therapy	G-CSF SD	23	NA	NA
		Pegfilgrastim 12 mg	22	NA	NA
		Pegfilgrastim 18 mg	22	NA	NA
Czerw et al. [30]	MM patients, age 18–65 years, CR or PR achieved after at least one line of therapy	G-CSF SD	46	60 (37–65)	57%
		ID-AraC + G-CSF SD	44	56 (33–65)	61%
DiPersio et al. [33]	Diagnosis of MM, age 18–78 years, in first or second CR or PR	G-CSF SD	154	58.4 ± 8.6	68%
		G-CSF SD + Plerixafor SD	148	58.2 ± 8.4	70%
Nahi et al. [50]	Diagnosis of MM, age ≥ 18 years, in CR or PR	G-CSF SD	10	58 (42–69)	60%
		G-CSF SD + Plerixafor SD	10	59 (43–70)	40%
Ri et al. [55]	Diagnosis of MM, age 20–75 years, in first or second CR or PR	G-CSF SD	7	60 (49–67)	57%
		G-CSF SD + Plerixafor SD	7	60 (38–71)	57%
Silvennoinen et al. [58]	Transplant-eligible MM patients aged ≤ 70 years	G-CSF SD	35	63 (40–70)	54%
		CY + G-CSF RD	34	62 (48–69)	53%
Skopec et al. [59]	Newly diagnosed MM treated with 3–6 cycles of Bor and Dex	G-CSF SD	20	60 (35–69)	55%
		Pegfilgrastim 12 mg	19	64 (51–71)	47%
Valtola et al. [61]	Transplant-eligible MM patients less than 70 years of age	G-CSF SD	19	63 (52–70)	42%
		CY + G-CSF RD	17	58 (49–70)	59%
**Studies for NHL**					
DiPersio et al. [32]	Diagnosis of NHL, age 18–78 years, in first or second CR or PR	G-CSF SD	148	59 (22–75)	69%
		G-CSF SD + Plerixafor SD	150	56 (29–75)	67%
Kuruvilla et al. [43]	Diagnosis of NHL, age 18–78 years, in first or second CR or PR	G-CSF SD + Plerixafor SD	31	47.8 ± 13.6	55%
		G-CSF SD + Plerixafor FD	30	46.1 ± 13.4	60%
Liu et al. [44]	NHL patients, age 18–65 years, achieving CR or PR after first- or second-line therapy	G-CSF SD	50	50 (18–64)	50%
		G-CSF SD + YF-H-2015005	51	45 (18–65)	53%
Matsue et al. [48]	Diagnosis of NHL, age 20–75 years, in first CR or PR	G-CSF SD	16	63 (27–70)	75%
		G-CSF SD + Plerixafor SD	16	56 (39–73)	69%
Zhu et al. [66]	Diagnosis of NHL, age 18–75 years, in first or second CR or PR	G-CSF SD	50	43 (20–60)	52%
		G-CSF SD + Plerixafor SD	50	39 (18–66)	62%

Bor, bortezomib; CR, complete remission; CY, cyclophosphamide; Dex, dexamethasone; FD, fixed dose; G-CSF, granulocyte colony-stimulating factor; HL, Hodgkin lymphoma; ID-AraC, intermediate-dose cytarabine; MM, multiple myeloma; NA, not available; NHL, non-Hodgkin lymphoma; PR, partial remission; RD, reduced dose; SD, standard dose; YF-H-2015005, a new CXCR4 antagonist

*Age is presented as mean and range, or mean ± standard deviation

### The number of collected CD34 + cells

**Fig. 2:**
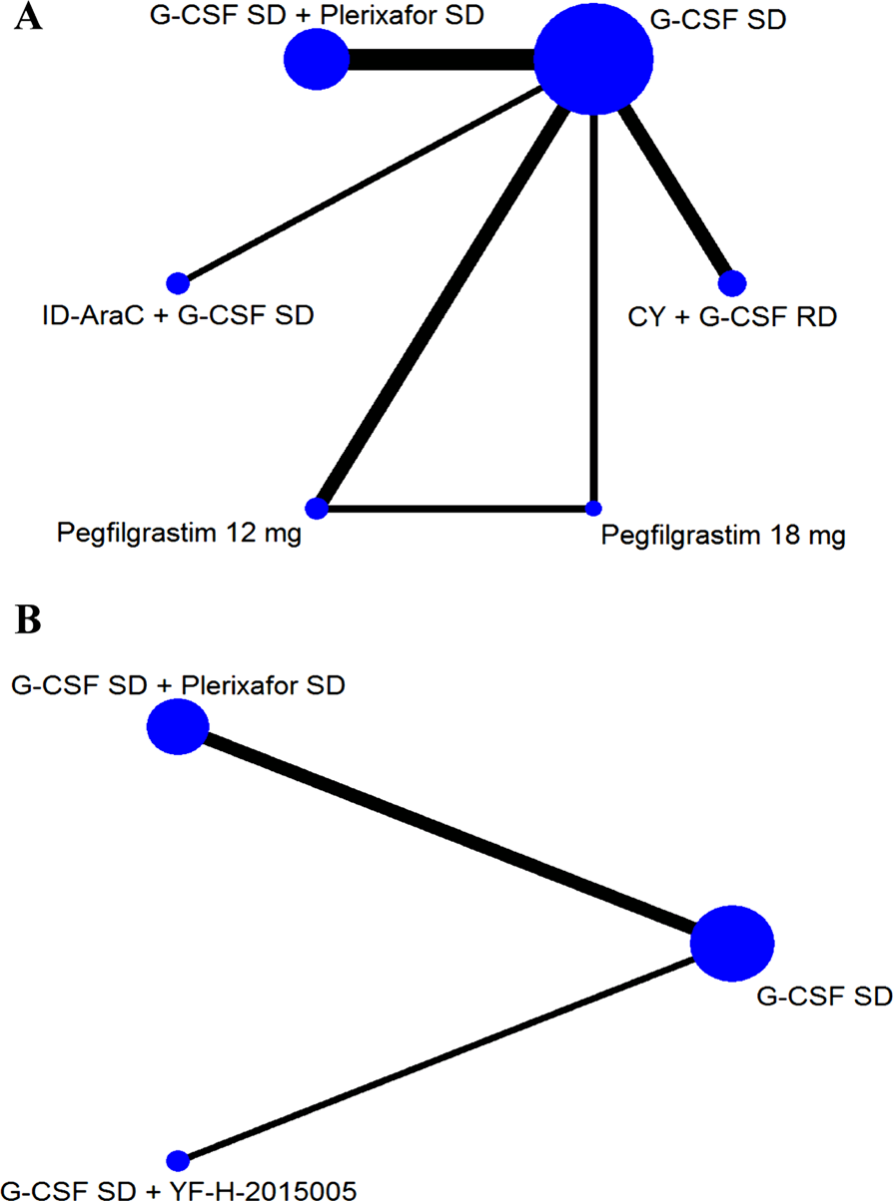
Network plot for total number of collected CD34^+^ cells. Network plot depicting all direct comparisons in included trials with data about the total number of collected CD34^+^ cell (× 10^6^/kg) for patients with MM (**A**) and NHL (**B**). Each node represents a mobilization regimen, while each line represents direct comparison between two regimens, with the thickness reflecting the number of times of direct comparisons. CY, cyclophosphamide; G-CSF, granulocyte colony-stimulating factor; ID-AraC, intermediate-dose cytarabine; RD, reduced dose; SD, standard dose; YF-H-2015005, a new CXCR4 antagonist

**Fig. 3:**
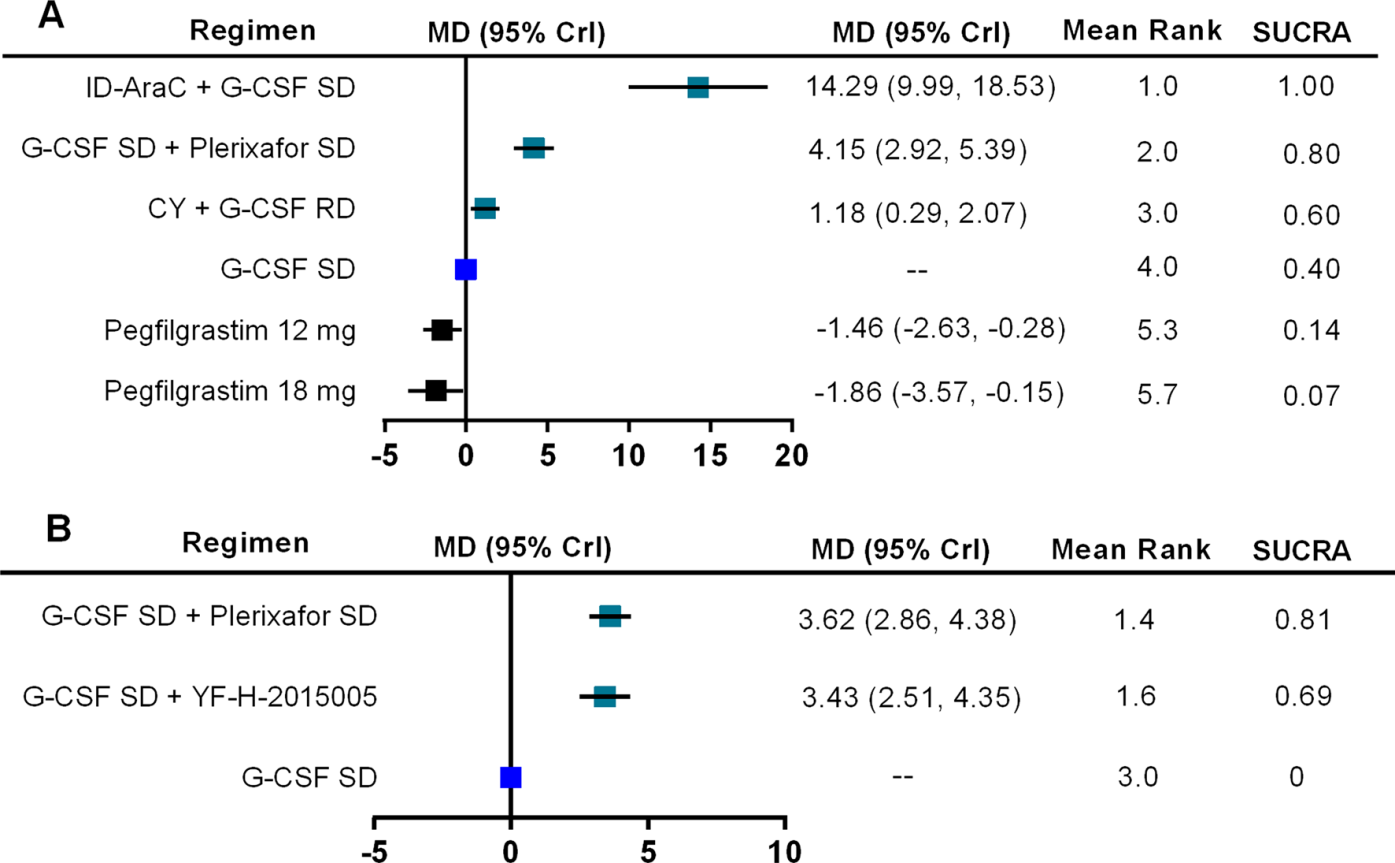
Forest plot of meta-analysis results for the total number of collected CD34^+^ cells. Forest plot regarding the network meta-analysis results of the total number of collected CD34^+^ cells (× 10^6^/kg) for patients with MM (**A**) and NHL (**B**). G-CSF SD is the common comparator. Estimate of relative treatment effect for other mobilization regimens are reported as mean differences (MD) with the associated 95% credibility interval (95% CrI). CY, cyclophosphamide; G-CSF, granulocyte colony-stimulating factor; ID-AraC, intermediate-dose cytarabine; RD, reduced dose; SUCRA, surface under the cumulative ranking curve; SD, standard dose; YF-H-2015005, a new CXCR4 antagonist

The total number of CD34^+^ cells (× 10^6^/kg) collected from PB are reported in 8 trials for MM, involving 6 mobilizaion regimens. The network plot for all direct comparisons is shown in Fig. 2A. Results of network meta-analysis using fixed-effects model show that compared with G-CSF SD alone, 3 regimens including ID-AraC + G-CSF SD (MD 14.29, 95% CrI 9.99–18.53; SUCRA 1.00), G-CSF SD + Plerixafor SD (MD 4.15, 95% CrI 2.92–5.39; SUCRA 0.80), and CY + G-CSF RD (MD 1.18, 95% CrI 0.29–2.07; SUCRA 0.60) are associated with significantly higher total number of CD34^+^ cells (× 10^6^/kg) collected.. Pegfilgrastim 12 mg and 18 mg are associated with lower number of CD34^+^ cells collected than G-CSF SD. The forest plot with MD and 95% CrI for all included regimens is shown in Fig. 3A. Regimens were ranked based on their relative treatment effects, ID-AraC + G-CSF SD ranking first with a probability of being best regimen of 100%. The SUCRA plots for all of the 6 regimens regarding the number of CD34^+^ cells collected in patients with MM are shown in Additional file 7: Figure S2.

As for patients with NHL, the number of CD34^+^ cells collected are compared among 3 regimens (Fig. 2B). Results of network meta-analysis using fixed-effects model show that compare with G-CSF SD, G-CSF SD + Plerixafor SD (MD 3.62, 95% CrI 2.86–4.38; SUCRA 0.81), and G-CSF SD + YF-H-2015005 (MD 3.43, 95% CrI 2.51–4.35; SUCRA 0.69) are associated with significantly higher total number of CD34^+^ cells (× 10^6^/kg) collected (Fig. 3B). The probabilities of G-CSF SD + Plerixafor SD and G-CSF SD + YF-H-2015005 being the best regimen are 62%, 38% respectively. The SUCRA plots for these 3 regimens regarding the number of CD34^+^ cells collected in patients with NHL are shown in Additional file 8: Figure S3.

### Successful mobilization rate

**Fig. 4:**
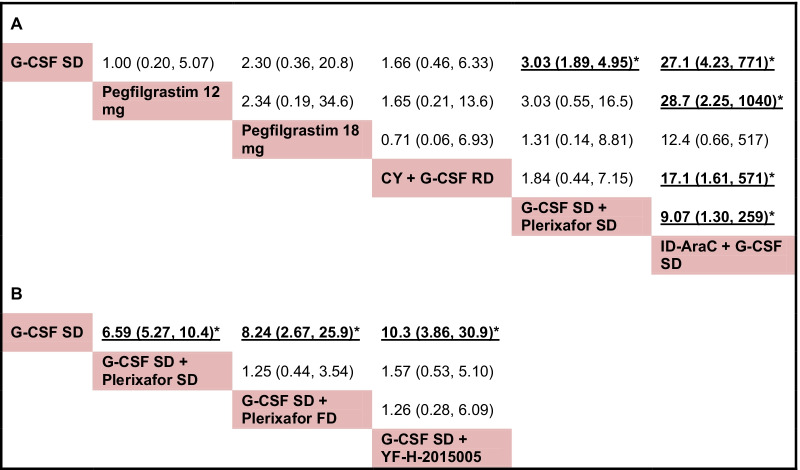
Pooled results for successful rate of achieving optimal target. Pooled ORs and 95% CrI for all possible head-to-head comparisons regarding the successful rate of achieving optimal target for patients with MM (**A**) and NHL (**B**). OR, odds ratio. 95% CrI, 95% credibility interval. CY, cyclophosphamide; FD, fixed dose; G-CSF, granulocyte colony-stimulating factor; ID-AraC, intermediate-dose cytarabine; RD, reduced dose; SD, standard dose; YF-H-2015005, a new CXCR4 antagonist

The successful rates of achieving optimal target (collecting ≥ 4–6 × 10^6^ CD34^+^ cells/kg) are compared among 6 mobilization regimens for patients with MM, the network plot describing all direct comparisons within these regimens is shown in Additional file 9: Figure S4A. Results of network meta-analysis using fixed-effects model suggest that compared with G-CSF SD alone, ID-AraC + G-CSF SD (OR 27.1, 95% CrI 4.23–771; SUCRA 0.99) and G-CSF SD + Plerixafor SD (OR 3.03, 95% CrI 1.89–4.95; SUCRA 0.66) are associated with significantly higher rate of achieving optimal target. In addition, ID-AraC + G-CSF SD is associated with significantly higher rate of achieving optimal target than Pegfilgrastim 12 mg, CY + G-CSF RD and G-CSF SD + Plerixafor SD. Other comparisons did not show any statistically significant results. Pooled ORs and the associated 95% CrI for all possible head-to-head comparisons are listed in Fig. 4A. ID-AraC + G-CSF SD ranked first with a probability of being best regimen of 94% in consideration the successful rate of achieving optimal target. The rank results and SUCRA values for all of the 6 regimens are provided in Additional file 5: Table S5. The SUCRA plots are shown in Additional file 10: Figure S5.

For patients with NHL, the successful rates of achieving optimal target are compared among 4 mobilization regimens (Additional file 9: Figure S4B). Network meta-analysis using fixed-effects model show that compared with G-CSF SD alone, G-CSF SD + Plerixafor SD (OR 6.59, 95% CrI 5.27–10.4; SUCRA 0.52), G-CSF SD + Plerixafor FD (OR 8.24, 95% CrI 2.67–25.9; SUCRA 0.68) and G-CSF SD + YF-H-2015005 (OR 10.3, 95% CrI 3.86–30.9; SUCRA 0.80) are associated increased rate of achieving optimal target. There is no significant difference between G-CSF SD + Plerixafor FD and G-CSF SD + Plerixafor SD, or between G-CSF SD + YF-H-2015005 and G-CSF SD + Plerixafor SD considering the successful rates of achieving optimal target (Fig. 4B). The rank results and SUCRA values for these 4 regimens are provided in Additional file 5: Table S5. The SUCRA plots are shown in Additional file 11: Figure S6.

## Discussion

This systematic review and network meta-analysis summarized the results of 44 RCTs comparing the efficacy of hematopoietic stem cell mobilization regimens in patients with hematological malignancies, and separately compared the efficacy of some regimens for patients with MM and NHL. We found that G-CSF SD + Plerixafor SD significantly improved hematopoietic stem cell mobilization efficacy compared with G-CSF SD alone both in patients with MM and NHL. In addition, ID-AraC + G-CSF SD also significantly improved mobilization efficacy in patients with MM, and it is associated with highest probability of being best regimen in consideration of both the number of total CD34^+^ cells collected and the successful rate of achieving optimal mobilization target. For patients with NHL, G-CSF SD plus a new CXCR4 antagonist YF-H-2015005 also significantly increased the number of total CD34^+^ cells collected and the successful rate of achieving optimal mobilization target. G-CSF SD + Plerixafor SD and G-CSF SD + YF-H-2015005 showed similar improvement in HSCs mobilization efficacy.

To the best of our knowledge, this is the first network meta-analysis comparing the efficacy of stem cell mobilization regimens in patients with hematological malignancies. Several traditional meta-analyses have been published in recent years, but they only could evaluate two regimens, such as G-CSF plus Plerixafor versus G-CSF alone, and pegylated G-CSF versus non-pegylated G-CSF [69–72] The relative effects of many other mobilization regimens are unclear due to the lack of direct comparison and integrated study. Our study overcome this limitation through pooling both direct and indirect evidences with network meta-analysis. We chose standard dose of G-CSF (10 μg/kg/day) as the common comparator since it remains the most commonly used mobilization regimen. The relative treatment effects of other mobilization regimens were estimated with well-established methods. Moreover, these regimens are ranked based on relative CD34^+^ cells yield and the successful rate of achieving optimal mobilization target. The SUCRA value and graphs for each regimen are provided to display the rank probabilities. Moreover, the ORs with associated 95% CrI regarding the successful rate of reaching optimal target for all head-to-head comparisons are provided. We consider that our results can facilitate regimen selection for patients with high risk of mobilization failure.

One of the most important findings of this study is that intermediate-dose cytarabine (ID-AraC) may be more efficient than cyclophosphamide (CY) when used for HSCs mobilization in patients with MM. Mobilizing chemotherapy followed by G-CSF is a commonly used mobilization strategy in patients with hematological malignancies. Although longer interval from drug administration to initiation of apheresis, higher risk of neutropenic fever, and increase require of hospitalization associated with chemotherapy-based mobilization are widely reported, patients still could greatly benefit from decreased tumor cells burden and high CD34^+^ cells yield [18]. For the past time, cyclophosphamide was the most commonly used mobilizing chemotherapeutic agent both in patients with MM. A meta-analysis including both prospective and retrospective studies suggested that cyclophosphamide 1–g/m^2^ plus G-CSF (RD or SD) is associated with significantly higher CD34^+^ cells yield in patients with MM when compared with G-CSF alone, which is consistent with our results [72]. A RCT reported by Czerw et al*.* suggested that ID-AraC plus G-CSF prominently increased CD34^+^ cells yield (median of 20.2 versus 5.9 × 10^6^/kg) compared with G-CSF alone, and produced a rate of achieving optimal target (≥ 5 × 10^6^ CD34^+^ cells/kg) with a single apheresis of 86% versus 41% [30]. A retrospective study suggested that ID-AraC plus G-CSF is more effective than CY plus G-CSF in HSCs mobilization in patients with MM [73]. Consistently, our results of indirect comparison suggested that ID-AraC + G-CSF SD is superior to CY + G-CSF RD. However, RCT that directly compare the mobilization efficacy of ID-AraC versus CY is unavailable. In addition, it is suggested that some countries commonly used another chemotherapeutic agent vinorelbine plus G-CSF as standard HSCs mobilizaion regimen for patients with MM [38, 57]. Well-designed RCTs comparing these regimens will be helpful to establish our results and determine the optimal mobilization regimens for MM patients.

As for patients with NHL, benefits of CY in HSCs mobilization are uncertain, the two RCTs enrolling participants before the year of 2000 to compare the efficacy of CY plus G-CSF versus G-CSF alone did not reach consistent conclusions [49, 51]. Nowadays, salvage therapy regimens such as DHAP, ESHAP, ICE and IEV (ifosphamide, epirubicin and etoposide) follow by G-CSF is a commonly used HSCs mobilization approach for patients with relapsed or refractory NHL, it eliminates the requirement of additional chemotherapy [74, 75]. A RCT comparing DHAP versus CY did not report significant difference in mean number of CD34^+^ cells collected [54]. RCTs comparing other salvage therapy regimens are not available. Retrospective studies comparing the mobilization efficacy of different chemotherapy regimens in NHL patients reported inconsistent results in different study design [75–77].Other mobilizing chemotherapy regimens including etoposide alone, ifosphamide alone, CE (cyclophosphamide, etoposide), CEP (cyclophosphamide, etoposide, and cisplatin), MEOD with or without MTX are also investigated, but the related trials failed to be integrated in our network meta-analysis due to the heterogeneity in study design and the lack of connection to the main network [29, 53, 62–64, 67]. In addition, mobilizing chemotherapy regimens varies across different disease subgroups and study centers. Therefore, the specific optimal mobilizing chemotherapy regimens for patients with NHL remain unclear due to the great heterogeneity in regimen components and the lack of direct comparison, further well-designed studies are required.

Moreover, our results demonstrated that G-CSF plus plerixafor or the new CXCR4 antagonist YF-H-2015005 improved mobilization efficiency. Since approved for stem cell mobilization by FDA in 2008, the CXCR4 antagonist plerixafor exhibited favorable mobilization results in patients with NHL and MM [32, 33]. Superiority of plerixafor plus G-CSF versus placebo plus G-CSF has been well established in series of RCTs, but RCT that directly comparing plerixafor plus G-CSF versus chemotherapy plus G-CSF are not available. By integrating both direct and indirect evidence, our network meta-analysis shows that plerixafor plus G-CSF SD is inferior to ID-AraC plus G-CSF SD, but superior to CY plus G-CSF RD in consideration of the rank probabilities regarding to the number of collected CD34^+^ cells and the rate of successful mobilization in patients with MM. Several retrospective studies suggested that plerixafor plus G-CSF is associated with comparable or lower CD34^+^ cells yield, but lower risk of neutropenic fever, reduced need of antibiotics use and unscheduled hospitalization compared with cyclophosphamide plus G-CSF SD in patients with MM [78–80]. However, mobilization with plerixafor is associated with potentially high economic cost, hence risk-adapted strategies in which plerixafor is only used to patients with high risk of mobilization failure, and salvage strategies in which plerixafor is administrated to patients failed to prior mobilization are recommended [13, 81]. As for patients with NHL, chemotherapy-based mobilization using ICE plus G-CSF showed superior mobilization efficacy and comparable toxicity profile than plerixafor-based mobilization in a retrospective study, the efficacy and safety of other chemotherapy regimens versus plerixafor required to be investigated with further research [82]. The efficacy and safety of new CXCR4 antagonists including YF-H-2015005 and BL-8040 for mobilization are also investigated. Results of the RCT conducted by Liu et al*.* suggested that YF-H-2015005 plus G-CSF can significantly improving mobilization efficacy compared with placebo plus G-CSF in patients with NHL [44]. Our results of indirect comparison suggedted YF-H-2015005 and plerixafor are associated with similar HSCs mobilization efficacy. The Phase III RCT assessing the superiority of BL-8040 plus G-CSF versus placebo plus G-CSF still have no published data when this manuscript is completed [83]. We previously compared the mobilization efficacy of G-CSF alone, G-CSF plus plerixafor (AMD3100) and new regimens with a network meta-analysis of preclinical studies, and the results suggested that G-CSF plus plerixafor still has stable advantages even when several new CXCR4 antagonists and many new agents of different mechanisms have been developed [84]. In addition, most of the new CXCR4 antagonists and new agents of other mechanisms have not been used in humans. Before the superiority and safety profiles of these new CXCR4 antagonists are well established, plerixafor remains the preferred choice for risk-adapted mobilization and salvage mobilization.

The HSCs mobilization potentials of other hematopoietic growth factors such as TPO, GM-CSF, SCF and EPO are also reviewed in this study. TPO is considered as an attractive agent to be used in combination with chemotherapy plus G-CSF in HSCs mobilization [67]. Recombinant human thrombopoietin (rhTPO) is a full-length glycosylated molecule that can stimulate thrombocytopoiesis via activating the cytokine receptor c-Mpl [85]. Series of studies with different design have reported that rhTPO in combination with chemotherapy and G-CSF prominently enhanced PBSCs mobilization in patients with breast cancer, lymphoma and MM [86–88]. In the RCT performed by Zhu et al*.*, 15,000 U of rhTPO plus G-CSF and mobilizing chemotherapy led to approximately two-fold increase in CD34^+^ cells yields and proportions of patients reaching optimal target without increased toxicity compared with the non-TPO group [67]. However, this study failed to be included in our network meta-analysis due to the lack of connection, more studies are still required to establish the benefit role of TPO because of the limited number of published randomized trials and lack of integrated study comparing mobilization regimens with or without TPO. As for other growth factors such as GM-CSF and EPO, RCTs showed that the increase of collected CD34^+^ cells resulted from adding these factors are not statistically significant [36, 45, 60]. Although the GM-CSF alone or in combination with chemotherapy can induce HSCs mobilization, the use of GM-CSF-based mobilization in recent years is limited since it is associated with lower CD34^+^ cells yield, increased toxicity and delayed platelet recovery compared with G-CSF [24, 31, 89]. However, it is hypothesized GM-CSF-mobilized grafts are associated with enhanced immune reconstitution and lower risk of graft-versus-host disease (GVHD) after allogenic transplantation due to the differences in subsets of T cells and dendritic cells [90, 91]. Therefore, the role of GM-CSF in stem cells mobilization still require to be established with further investigations. As for SCF, although the included RCTs suggestd that SCF plus G-CSF is not significantly superior to G-CSF alone and CY plus G-CSF, several retrospective studies that carried out in poor mobilizer suggested that it could be an alternative regimen for patients failed to mobilization with G-CSF alone [39, 60, 92, 93]. Evidence for another growth factor EPO is limited. Study of Hart et al*.* suggested that EPO in combination with G-CSF and chemotherapy mildly increased CD34^+^ cells yields and reduced requirement of supportive therapy after transplantation, but the sample size is small [36]. To the best of our knowledge, until now, G-CSF and GM-CSF are the only two cytokines that has been approved for stem cell mobilization by the United States (US) Food and Drug Administration (FDA) [12]. Recombinant human SCF ancestim is approved in Canada and New Zealand, but not available in the US and seldom used in Europe due to the increased risk of side effects [14, 16]. Therefore, the selection of cytokines for stem cell mobilization should take the availability and toxicity into consideration in addition to mobilization efficiency.

As for the most commonly used HSCs mobilizing agent G-CSF, different forms are available now, including the non-glycosylated G-CSF filgrastim, glycosylated G-CSF lenograstim, pegylated G-CSF pegfilgrastim and G-CSF biosimilars. In this study, we integrated data from the two different forms of G-CSF (filgrastim and lenograstim) together, because RCTs did not show any significant difference between filgrastim and lenograstim in terms of mobilization efficacy in both healthy donors and patients with hematological malignancies [52, 68, 94]. Other G-CSF variants including biosimilar G-CSF and pegfilgrastim showed comparable mobilization efficacy, similar toxicity profile and reduced cost in comparison to G-CSF originator according to results of our meta-analysis and previously published studies [69, 95]. Pegfilgrastim provides a convenient alternative to filgrastim due to its extended half-life, a single dose of pegfilgrastim can achieve similar effects of repeated-dose G-CSF [69, 70]. Taken together, the mobilization efficacy of different forms of G-CSF are comparable.

There are several limitations in our study. First of all, as mentioned above, some chemotherapy-based mobilization regimens such as vinorelbine plus G-CSF for MM patients and different salvage therapy regimens plus G-CSF for NHL patients failed to be integrated in our network meta-analysis due to the heterogeneity in study design and the lack of connection to the main network. The HSCs mobilization efficacy of these regimens are carefully reviewed in Table 1 and Additional file 3: Table S3. Our results provide indirect evidence that cyclophosphamide alone is not always the best options for mobilizing chemotherapy, but the specific optimal mobilizing chemotherapy regimens for patients with different diagnosis remain unclear, futher well-designed trials directly comparing the efficacy of specific chemotherapy regimens will be helpful to solve this problem. Secondly, supportive evidence for some regimens (i.e., ID-AraC, YF-H-2015005) were derived from a limited number of studies. Our study is the first network meta-analysis which investigate the mobilizing efficacy of different regimens in human beings, in the future, the results of this meta-analysis could be updated by including data from newly reported trials. Thirdly, subgroup results for patients with different characteristics are not available due to the lack of subgroup data in included studies. Patients with NHL and MM differs in HSCs mobilization strategies and outcomes, and patients with NHL are associated with higher risk of mobilization failure, so we perform analysis separately for NHL and MM in this study [96]. Other factors such as advanced age, previous extensive chemotherapy, complicated with diabetes mellitus and smoking history are also associated with increased risk of mobilization failure [13]. Further studies are required to determine the optimal risk-adapted mobilization strategies in patients with different baseline characteristics. Lastly, publication bias can not be ruled out in this study, the interpretation of our results should be in cautions.

## Conclusions

In conclusion, our study summarized the results of 44 RCTs comparing different hematopoietic stem cell mobilization regimens for patients with hematological malignancies and compared the efficacy of mobilization regimens separately for patients with MM and NHL. ID-AraC plus G-CSF is associated with the highest probability of being best mobilization regimen in patients with MM. For patients with NHL, G-CSF in combination with plerixafor or YF-H-2015005 significantly improved HSCs mobilization efficacy, salvage therapy regimen followed by G-CSF is also a widely used mobilization strategy but the optimal salvage therapy regimens for mobilization in different disease subtypes still require to be establish with further research.

## Supplementary Information

**Additional file 1: Table S1.** Detailed information of all mobilizing chemotherapy regimens.

**Additional file 2: Table S2.** The specific dosage of mobilization regimens in all included studies.

**Additional file 3: Table S3.** The characteristics and mobilization results of the 44 trials included in review.

**Additional file 4: Table S4.** Results of risk of bias assessment.

**Additional file 5: Table S5.** Results of meta-analysis regarding the rate of reaching optimal target.

**Additional file 6: Figure S1.** The comparison-adjusted funnel plot. A, G-CSF SD; B, Pegfilgrastim 12 mg; C, Pegfilgrastim 18 mg; D, ID-AraC + G-CSF SD; E, G-CSF SD + Plerixafor SD; F, CY + G-CSF RD; G, G-CSF SD + Plerixafor FD; H, G-CSF SD + YF-H-2015005.

**Additional file 7: Figure S2.** The SUCRA graphs regarding the number of total CD34^+^ cells collected for patients with MM.

**Additional file 8: Figure S3.** The SUCRA graphs regarding the number of total CD34^+^ cells collected for patients with NHL.

**Additional file 9: Figure S4.** Network plot depicting all direct comparisons in included trials with data about the successful rate of reaching optimal target for patients with MM (A) and NHL (B).

**Additional file 10: Figure S5.** The SUCRA graphs regarding the successful rate of reaching optimal target for patients with MM.

**Additional file 11: Figure S6.** The SUCRA graphs regarding the successful rate of reaching optimal target for patients with NHL.

## Data Availability

All supporting data are included in the article and its additional files.

## Abbreviations

ASCT: Autologous hematopoietic stem cell transplantation
BL-8040: A new CXCR4 antagonist
CE: Cyclophosphamide, etoposide
CEP: Cyclophosphamide, etoposide, and cisplatin
CXCR-4: CXC chemokine receptor-4
CY: Cyclophosphamide
DHAP: Dexamethasone, high-dose cytarabine, and cisplatin
EPO: Erythropoietin
ESHAP: Etoposide, methylprednisolone, high-dose cytarabine, and cisplatin
FD: Fixed dose
FDA: Food and Drug Administration
G-CSF: Granulocyte colony-stimulating factor
GM-CSF: Granulocyte–macrophage colony-stimulating factor
HL: Hodgkin lymphoma
HDT: High-dose chemotherapy
HSCs: Hematopoietic stem cells
ICE: Ifosfamide, carboplatin and etoposide
ID-AraC: Intermediate-dose cytarabine
IEV: Ifosfamide, epirubicin and etoposide
IL-11: Interleukin 11
MA: Methotrexate, cytarabine
MD: Mean differences
MEOD: Mitoxantrone, etoposide, vindesine and dexamethasone
MM: Multiple myeloma
MTX: Methotrexate
NHL: Non-Hodgkin lymphoma
OR: Odds ratio
PBSCs: Peripheral blood stem cells
RCTs: Randomized controlled trials
RD: Reduced dose
SCF: Stem cell factor
SD: Standard dose
SDF-1: Stromal derived factor-1
TPO: Thrombopoietin
YF-H-2015005: A new CXCR4 antagonist
95% CrI: 95% Credibility interval
