# Supplementary material for: Efficacy of hematopoietic stem cell mobilization regimens in patients with hematological malignancies: a systematic review and network meta-analysis of randomized controlled trials
Source: Stem Cell Res Ther. 2022 Mar 22;13:123. doi: 10.1186/s13287-022-02802-6 (PMC8939102; doi:10.1186/s13287-022-02802-6)
Supplement: Supplementary file 1 — Additional file 1: Table S1. Detailed information of all mobilizing chemotherapy regimens. [file 13287_2022_2802_MOESM1_ESM.docx]

**Supplementary Table 1. Detailed information of all mobilizing chemotherapy regimens.**

| **Regimen** | **Dosage** | **Patients** | **Reference** | |
| --- | --- | --- | --- | --- |
| ***Single chemotherapeutic agents*** | | | |  |
| CY | CY 2-7 g/m^2^ i.v. on day 1 or on 2 successive days. | MM | Bourin 2004 [27], Demuynck 1995 [31], Facon 1999 [34], Karanth 2004 [40], Kim 2005 [41], Kuan 2015 [42], Orciuolo 2011 [52], Samaras 2018 [57], Silvennoinen 2016 [58], Vela-Ojeda 2000 [62] | |
|  |  | NHL | Chen 2010 [28], Gazitt 2001 [35], Johnsen 2011 [39], Karanth 2004 [40], Kuan 2015 [42], Milone 2003 [49], Narayanasami 2001 [51], Pavone 2002 [54], Vela-Ojeda 2000 [62] | |
|  |  | HL | Johnsen 2011 [39], Karanth 2004 [40], Kuan 2015 [42], Milone 2003 [49], Narayanasami 2001 [51], Vela-Ojeda 2000 [62] | |
| Etoposide | Etoposide (VP-16) 2 g/m^2^ i.v. over 4 h on day 0. | NHL | Copelan 2009 [29] | |
| Gemcitabine | Gemcitabine 1250 mg/m^2^ i.v. on day 1. | MM | Jeker 2020 [38] | |
| ID Ara-C | Cytarabine 4 g/m^2^ i.v. twice daily on days 1-2 (total dose 1.6 g/m^2^). | MM | Czerw 2019 [30], Manko 2014 [46] | |
| Ifosfamide | Ifosfamide 10 g/m^2^ i.v. over 2 h, divided on 2 successive days. | NHL, HL and MM | Vela-Ojeda 2000 [62] | |
| Vinorelbine | Vinorelbine 35 mg/m^2^ i.v. on day 1. | MM | Jeker 2020 [38], Samaras 2018 [57] | |
| ***Combined chemotherapy regimens*** | | | |  |
| CE | Cyclophosphamide 4 g/m^2^ i.v. on day 1, or 2 g/m^2^ i.v. on days 1-2; Etoposide (VP-16) 200 mg/m^2^/day i.v. on day 1 or days 1-3. | NHL, HL and MM | Lonial 2004 [45], Ozcelik 2009 [53], Weaver 1998 [63], Zhu 2019 [67] | |
| CEP | Cyclophosphamide 4 g/m^2^ i.v. on day 1; Etoposide 200 mg/m^2^/day i.v. on days 1-3; Cisplatin 35 mg/m^2^ on days 1-3. | NHL and HL | Weaver 1998 [63] | |
| CMD | Cyclophosphamide 4 g/m^2^ i.v. on day 1; Mitoxantrone 8 g/m^2^ i.v. on day 1-2; Dexamethasone 20 mg/m^2^ i.v. every 12 hours on day 1-2. | MM | Arora 2004 [24] | |
| DHAP | Cisplatin 100 mg/m^2^ i.v. over 24h; Cytarabine in two doses of 2 g/m^2^ given 12 h apart; Dexamethasone 40 mg i.v. on days 1-4. | NHL | Pavone 2002 [54] | |
| ESHAP | Etoposide 40 mg/m^2^ i.v. on days 1-4; Cisplatin 25 mg/m^2^ i.v. on days 1-4; Cytarabine 2 g/m^2^ i.v. on day 5; Methylprednisolone 500 mg i.v. on days 1-5. | NHL | Kim 2005 [41], Manko 2014 [46] | |
| ICE | Ifosfamide 5 g/m^2^ i.v. on day 2, Carboplatin AUC 5 i.v. on day 2, Etoposide 100 mg/m^2^/day i.v. on days 1-3. | NHL | Lonial 2004 [45], Russell 2008 [56] | |
| IEV | Ifosfamide 2.5 g/m^2^, Epirubicin 100 mg/m^2^, Etoposide (VP-16) 150 mg/m^2^ i.v. on days 1-3. | MM | Hart 2009 [36] | |
| MA | Methotrexate 1g/m^2^ i.v. on day 1; Cytarabine 2 g/m^2^/12h i.v. on days 2-3. | NHL | Chen 2010 [28] | |
| MEOD | Mitoxantrone 12 mg/m^2^ given as a split dose over 3 days; Etoposide 100 mg/day for 3 days; Vindesine 3 mg/m^2^ for 1 day; Dexamethasone 10 mg/day for 7 days. | NHL | Zhang 2014 [64] | |
| MEOD + MTX | MEOD; MTX 2 g/m^2^ for 1 day. | NHL | Zhang 2014 [64] | |

Abbreviation: CY, cyclophosphamide; CE, cyclophosphamide and etoposide; CEP, cyclophosphamide and etoposide plus cisplatin; CMD, cyclophosphamide, mitoxantrone and dexamethasone; DHAP, dexamethasone, high-dose cytarabine, and cisplatin; ESHAP, etoposide, methylprednisolone, high-dose cytarabine, and cisplatin; HL, Hodgkin lymphoma; ICE, ifosfamide, carboplatin and etoposide; ID-AraC, intermediate-dose cytarabine; IEV, ifosfamide, epirubicin and etoposide; MA, methotrexate, cytarabine; MEOD, mitoxantrone, etoposide, vindesine and dexamethasone; MM, multiple myeloma; MTX, methotrexate; NHL, non-Hodgkin lymphoma; VP-16, etoposide.
