# Supplementary material for: Efficacy of hematopoietic stem cell mobilization regimens in patients with hematological malignancies: a systematic review and network meta-analysis of randomized controlled trials
Source: Stem Cell Res Ther. 2022 Mar 22;13:123. doi: 10.1186/s13287-022-02802-6 (PMC8939102; doi:10.1186/s13287-022-02802-6)
Supplement: Supplementary file 2 — Additional file 2: Table S2. The specific dosage of mobilization regimens in all included studies. [file 13287_2022_2802_MOESM2_ESM.docx]

**Supplementary Table 2. The specific dosage of mobilization regimens in all included studies.**

| Study | Study arm | Dosage |
| --- | --- | --- |
| Arora 2004 [24] | CMD + G-CSF RD | 2 cycles of CMD (CY 4 g/m^2^ i.v. on day 1, Mitoxantrone 8 g/m^2^ i.v. on days 1-2, Dexamethasone 20 mg/m^2^ i.v. every 12 hours on days 1-2); G-CSF 250 μg/m^2^/day s.c. starting on day 4 until PBSC collections were complete (2-3 days). |
|  | CMD + GM-CSF RD | 2 cycles of CMD; GM-CSF 250 μg/m^2^/day s.c. starting on day 4 until PBSC collections were complete (2-3 days). |
| Bhamidipati 2017 [25] | Biosimilar G-CSF + Plerixafor SD | Biosimilar G-CSF (Tbo-Filgrastim) 10 μg/kg/day s.c. on days 1-5; Plerixafor 0.24 mg/kg s.c. on day 4; Biosimilar G-CSF + Plerixafor was repeated up to 4 times until the target collection goal was met. |
|  | G-CSF SD + Plerixafor SD | G-CSF (Filgrastim) 10 μg/kg/day s.c. on days 1-5; Plerixafor 0.24 mg/kg s.c. on day 4; G-CSF + Plerixafor was repeated up to 4 times until the target collection goal was met. |
| Bouko 2013 [26] | G-CSF SD | G-CSF (Filgrastim) 10 μg/kg/day administered until last day of leukapheresis. |
|  | Pegfilgrastim 12 mg | A single dose of Pegfilgrastim 12 mg. |
|  | Pegfilgrastim 18 mg | A single dose of Pegfilgrastim 18 mg. |
| Bourin 2004 [27] | CY + G-CSF RD | CY 4 g/m^2^; G-CSF 5 μg/kg/day. |
|  | SCF + G-CSF SD | SCF 25 μg/kg/day; G-CSF 5 μg/kg/day. |
| Chen 2010 [28] | CY + G-CSF RD | CY 2 g/m^2^ i.v. for 2 days; G-CSF 300 μg/day s.c. until the end of collection. |
|  | MA + G-CSF RD | MA (MTX 1g/m^2^ i.v. on day 1; Ara-c 2 g/m^2^/12h i.v. on days 2-3); G-CSF 300 μg/day s.c. until the end of collection |
| Copelan 2009 [29] | VP-16 + G-CSF SD | Etoposide (VP-16) 2 g/m^2^ i.v. on day 0; G-CSF 10 μg/kg/day s.c. starting on day 2. |
|  | Rituximab + VP-16 + G-CSF SD | Rituximab 375 mg/m^2^ i.v. on days -14, -7 and - 1; Etoposide 2 g/m^2^ i.v. on day 0; G-CSF 10 μg/kg/day s.c. starting on day 2. |
| Czerw 2019 [30] | G-CSF SD | G-CSF (Filgrastim) 10 μg/kg/day s.c. for up to 7 days. |
|  | ID-AraC + G-CSF SD | ID-AraC 0.4 g/m^2^ i.v. twice daily on days +1 and +2 (total dose 1.6 g/m^2^); G-CSF (Filgrastim) 10 μg/kg/day s.c. started on day +5. |
| Demuynck 1995 [31] | CY + G-CSF SD | CY 4g/m^2^ i.v. on day 0; G-CSF 10 μg/kg/day s.c. initiated at day 4 and continued until completion of collection. |
|  | CY + GM-CSF SD | CY 4g/m^2^ i.v. on day 0; GM-CSF 10 μg/kg/day s.c. initiated at day 4 and continued until completion of collection. |
| DiPersio 2009-3101 [32] | G-CSF SD + Plerixafor SD | G-CSF 10 μg/kg/day s.c. in the morning for up to 8 days; Plerixafor 0.24 mg/kg/day s.c. beginning on the evening of day 4 for up to 4 days. |
|  | G-CSF SD | G-CSF 10 μg/kg/day s.c. in the morning for up to 8 days; Placebo s.c. beginning on the evening of day 4 for up to 4 days. |
| DiPersio 2009-3102 [33] | G-CSF SD + Plerixafor SD | G-CSF 10 μg/kg/day s.c. in the morning for up to 8 days; Plerixafor 0.24 mg/kg/day s.c. beelining on the evening of day 4 for up to 4 days. |
|  | G-CSF SD | G-CSF 10 μg/kg/day s.c. in the morning for up to 8 days; Placebo s.c. beginning on the evening of day 4 for up to 4 days. |
| Facon 1999 [34] | CY + SCF + G-CSF RD | CY 4 g/m^2^ i.v. on day 1; SCF 20 μg/kg/day, G-CSF (Filgrastim) 5 μg/kg/day s.c. starting on day 2 until all leukaphereses were completed |
|  | CY + G-CSF RD | CY 4 g/m^2^ i.v. on day 1; G-CSF (Filgrastim) 5 μg/kg/day s.c. starting on day 2 until all leukaphereses were completed. |
| Gazitt 2001 [35] | CY + G-CSF SD | CY 3 g/m^2^; G-CSF 10 μg/kg/day. |
|  | CY + GM-CSF RD | CY 3 g/m^2^; GM-CSF 250 μg/m^2^/day. |
|  | CY + GM-CSF RD + G-CSF SD | CY 3 g/m^2^; GM-CSF 250 μg/m^2^/day for 7 days; G-CSF 10 μg/kg/day for 8 days. |
| Hart 2009 [36] | IEV + G-CSF RD | IEV (Ifosfamide 2500 mg/m^2^, Etoposide 150 mg/m^2^ and Epirubicin 100 mg/m^2^ i.v. on days 1-3); G-CSF (Filgrastim) 5 μg/kg/day s.c. twice daily starting on the third day after chemotherapy until the last day of leukapheresis. |
|  | IEV + G-CSF RD + EPO | IEV; G-CSF (Filgrastim) 5 μg/kg/day s.c. twice daily starting on the third day after chemotherapy until the last day of leukapheresis; recombinant human EPO-α 150 IE/kg/day s.c. beginning at day 1 after chemotherapy until the collection was completed. |
| Hohaus 1998 [37] | G-CSF RD | G-CSF (Filgrastim) 5 μg/kg/day s.c. 1 day after the end of cytotoxic chemotherapy until the last day of leukapheresis. |
|  | GM-CSF RD | GM-CSF (Molgramostim) 5 μg/kg/day s.c. 1 day after the end of cytotoxic chemotherapy until the last day of leukapheresis. |
| Jeker 2020 [38] | Vinorelbine + G-CSF SD | Vinorelbine 35 mg/m^2^ i.v. at day 1; G-CSF 60-96 Mio I.U./day s.c. staring on day 4 until the day of collection. |
|  | Gemcitabine + G-CSF SD | Gemcitabine 1250 mg/m^2^ i.v. at day 1; G-CSF 60-96 Mio I.U./day s.c. staring on day 4 until the day of collection. |
| Johnsen 2011 [39] | CY + G-CSF SD | CY 2-4 g/m^2^ i.v. on day 0; G-CSF (Filgrastim) 10 μg/kg/day s.c. started on day 1 until leukaphereses have been completed. |
|  | SCF + G-CSF SD | G-CSF (Filgrastim) 10 μg/kg/day, SCF 20 μg/kg/day s.c. started on day 1 until leukaphereses have been completed. |
| Karanth 2004 [40] | CY + G-CSF RD | CY 2 g/m^2^ i.v. on day 1; G-CSF (Lenograstim) 5 μg/kg/day administrated on days 1-5. |
|  | G-CSF SD | G-CSF (Lenograstim) 10 μg/kg/day administrated on days 1-5. |
| Kim 2005 [41] | CY or ESHAP ± Rituximab + G-CSF SD (single dose) | Disease-based mobilizing chemotherapy (CY 4 g/m2 for MM, ESHAP with or without Rituximab for NHL); G-CSF (Lenograstim) 10 μg/kg once a day starting on the day after completion of mobilization chemotherapy and continued until the last leukapheresis. |
|  | CY or ESHAP ± Rituximab + G-CSF SD (split dose) | Disease-based mobilizing chemotherapy; G-CSF (Lenograstim) 5 μg/kg twice a day starting on the day after completion of mobilization chemotherapy and continued until the last leukapheresis. |
| Kuan 2015 [42] | CY + G-CSF RD | CY 2 g/m^2^ i.v. on day 1; G-CSF (Filgrastim) 5 μg/kg/day s.c. on days 3-7. |
|  | CY + Pegfilgrastim 6 mg | CY 2 g/m^2^ i.v. on day 1; Pegfilgrastim single dose of 6 mg s.c. on day 3. |
|  | CY + late Pegfilgrastim 6 mg | CY 2 g/m^2^ i.v. on day 1; Pegfilgrastim single dose of 6 mg s.c. on day 7. |
| Kuruvilla 2018 [43] | G-CSF SD + Plerixafor FD | G-CSF (Filgrastim) 10 μg/kg/day s.c. for 4 days; Plerixafor 20 mg s.c. on the evening of day 4; G-CSF + Plerixafor (Plerixafor administration in the evening and G-CSF 1 h prior to apheresis in the following morning) was repeated for a maximum of 4 apheresis or until optimal target had been achieved. |
|  | G-CSF SD + Plerixafor SD | G-CSF (Filgrastim) 10 μg/kg/day s.c. for 4 days; Plerixafor 0.24 mg/kg s.c. on the evening of day 4; G-CSF + Plerixafor was repeated for a maximum of 4 apheresis or until optimal target had been achieved. |
| Liu 2021 [44] | G-CSF SD + YF-H-2015005 | G-CSF 10 μg/kg/day administrated in each morning for up to 8 days; YF-H-2015005 0.24 mg/kg/day starting on the evening of the day 4 and continued for up to 4 days. |
|  | G-CSF SD | G-CSF 10 μg/kg/day administrated in each morning for up to 8 days; Placebo 0.24 mg/kg/day starting on the evening of the day 4 and continued for up to 4 days. |
| Lonial 2004 [45] | Chemotherapy + G-CSF SD | CY (CY 4 g/m^2^ i.v.), CE (CY 4 g/m^2^, Etoposide 200 mg/m^2^ i.v.), or ICE (Ifosfamide 5 g/m^2^ i.v. on day 2, Carboplatin AUC 5 i.v. on day 2, Etoposide 100 mg/m^2^/day i.v. on days 1-3); G-CSF 5 μg/kg twice daily s.c. until the end of collection. |
|  | Chemotherapy + G-CSF RD + GM-CSF RD | CY, CE or ICE; G-CSF 5 μg/kg/day, GM-CSF 5 μg/kg/day s.c. until the end of collection. |
| Manko 2014 [46] | Chemotherapy + Biosimilar G-CSF SD | Etoposide, CY, ID-AraC or ESHAP; Biosimilar G-CSF (Zarzio) 10 μg/kg/day i.v. for a median of 8 days. |
|  | Chemotherapy + G-CSF SD | Etoposide, CY, ID-AraC or ESHAP; G-CSF (Filgrastim) 10 μg/kg/day |
| Marchesi 2018 [47] | Chemotherapy + G-CSF RD | Cytarabine-based, Ifosfamide-based, or Etoposide-based mobilizing chemotherapy; G-CSF (Lenograstim) 5 μg/kg/day s.c. starting on day +1 from the end of chemotherapy and continued until the end of collection. |
|  | Chemotherapy + Biosimilar G-CSF SD | Cytarabine-based, Ifosfamide-based, or Etoposide-based mobilizing chemotherapy; Biosimilar G-CSF 10 μg/kg/day s.c. starting on day +1 from the end of chemotherapy and continued until the end of collection. |
| Matsue 2018 [48] | G-CSF SD + Plerixafor SD | G-CSF 400 μg/m^2^/day s.c. for days 1-8; Plerixafor 240 μg/kg/day s.c. for days 4-7. |
|  | G-CSF SD | G-CSF 400 μg/m^2^/day s.c. for days 1-8 |
| Milone 2003 [49] | G-CSF SD | G-CSF (Lenograstim) 10 μg/kg/day s.c. in two daily doses from day +1 to the end of harvesting. |
|  | CY + G-CSF SD | CY 4 g/m^2^ i.v. on day +1; G-CSF (Lenograstim) 10 μg/kg/day s.c. in two daily doses from day +4. |
| Nahi 2019 [50] | G-CSF SD | G-CSF 10 μg/kg/day s.c. on days 1-5 |
|  | G-CSF SD + Plerixafor SD | G-CSF 10 μg/kg/day s.c. on days 1-5; Plerixafor 0.24 mg/kg/day s.c. on the evening of day 4, repeated on day 5-7 as needed. |
| Narayanasami 2001 [51] | G-CSF SD | G-CSF (Filgrastim) 10 μg//kg/day s.c. for 4 days. |
|  | CY + G-CSF SD | CY 5 g/m^2^ i.v.; G-CSF (Filgrastim) 10 μg//kg/day s.c. starting 24 hours late. |
| Orciuolo 2011 [52] | CY + G-CSF (Lenograstim) SD | CY 2-7 g/m^2^ i.v. on day 1; G-CSF (Lenograstim) 30-60 MU/day administrated from day 4 until the end of aphaeresis. |
|  | CY + G-CSF (Filgrastim) SD | CY 2-7 g/m^2^ i.v. on day 1; G-CSF (Filgrastim) 30-60 MU/day administrated from day 4 until the end of aphaeresis. |
| Ozcelik 2009 [53] | CE + ealy G-CSF SD | CE (CY 4 g/m^2^ on day 1, Etoposide 200 mg/m^2^/day on days 1-3); G-CSF 10 μg/kg/day i.v. twice daily started on day 4 after chemotherapy and continued until the last day of apheresis. |
|  | CE + late G-CSF SD | CE; G-CSF 10 μg/kg/day i.v. twice daily started on day 7 after chemotherapy and continued until the last day of apheresis. |
| Pavone 2002 [54] | DHAP + G-CSF RD | DHAP (Cisplatin 100 mg/m^2^ i.v., Ara-C in two doses of 2 g/m^2^ given 12 h apart, Dexamethasone 40 mg i.v. on days 1 to 4); G-CSF (Lenograstim) 5 μg/kg/day from day +2 after chemotherapy. |
|  | CY + G-CSF RD | CY 5 g/m^2^ given in five pulses each at a dose of 1 g/m^2^ by infusion over 1 h; G-CSF (Lenograstim) 5 μg/kg/day from day +2 after chemotherapy. |
| Ri 2017 [55] | G-CSF SD + Plerixafor SD | G-CSF (Filgrastim) 400 μg/m^2^/day s.c. on days 1-8; Plerixafor 240 μg/kg s.c. on the evening of days 4-7. |
|  | G-CSF SD | G-CSF (Filgrastim) 400 μg/m^2^/day s.c. on days 1-8. |
| Russell 2008 [56] | ICE + G-CSF RD | ICE (Etoposide 100 mg/m^2^ on days 1-3; Carboplatin AUC of 5 on day 2; Ifosfamide 5 g/m^2^ on day 2); G-CSF (Filgrastim) 5 µg/kg/day from day 4 until the last day of leukapheresis. |
|  | ICE + Pegfilgrastim 6 mg | ICE; Pegfilgrastim 6 mg on day 4. |
|  | ICE + Pegfilgrastim 12 mg | ICE; Pegfilgrastim 12 mg on day 4. |
| Samaras 2018 [57] | Vinorelbine + G-CSF SD | Vinorelbine 35 mg/m^2^ as a bolus infusion on day 1; G-CSF (Filgrastim) 5 μg/kg twice daily s.c. starting on day 4. |
|  | Vinorelbine + G-CSF RD | Vinorelbine 35 mg/m^2^ as a bolus infusion on day 1; G-CSF (Filgrastim) 5 μg/kg once daily s.c. starting on day 4. |
| Silvennoinen 2016 [58] | CY + G-CSF RD | CY 2 g/m^2^ on day +1; G-CSF (Filgrastim) 5 μg/kg/day starting on day +4. |
|  | G-CSF SD | G-CSF (Filgrastim) 10 μg/kg/day starting on day +1. |
| Skopec 2017 [59] | G-CSF SD | G-CSF (Filgrastim) 10 μg/kg/day s.c. from day 1 until collection was completed. |
|  | Pegfilgrastim 12mg | Pegfilgrastim 12 mg s.c. on day 1. |
| Stiff 2000 [60] | SCF + G-CSF SD | G-CSF (Filgrastim) 10 μg/kg/day, SCF 20 μg/kg/day s.c. for 5-9 days. |
|  | G-CSF SD | G-CSF (Filgrastim) 10 μg/kg/day s.c. for 5-9 days. |
| Valtola 2016 [61] | CY + G-CSF RD | CY 2 g/m^2^ i.v. on day +1; G-CSF (Filgrastim) 5 μg/kg/day from day +4. |
|  | G-CSF SD | G-CSF (Filgrastim) 10 μg/kg/day from day +1. |
| Vela-Ojeda 2000 [62] | Ifosfamide + GM-CSF RD | Ifosfamide 10 g/m^2^ i.v. over 2 h, divided on 2 successive days; GM-CSF 5 μg/kg/day s.c. started 24 h after completion of chemotherapy and continued until the last leukapheresis procedure. |
|  | CY + GM-CSF RD | CY 4 g/m^2^ i.v. over 2 h on 2 successive days; GM-CSF 5 μg/kg/day s.c. started 24 h after completion of chemotherapy and continued until the last leukapheresis procedure. |
| Weaver 1998 [63] | CE + G-CSF RD | CE (CY 4 g/m^2^ on day 1, Etoposide 200 mg/m^2^ on days 1-3); G-CSF 6 μg/kg/day from day 4 until the completion of apheresis. |
|  | CEP + G-CSF RD | CEP (CY 4 g/m^2^ on day 1, Etoposide 200 mg/m^2^ on days 1-3; Cisplatin 35 mg/m^2^ on days 1-3); G-CSF 6 μg/kg/day from day 4 until the completion of apheresis. |
| Zhang 2014 [64] | MEOD + G-CSF SD | MEOD (Mitoxantrone 12 mg/m^2^ given as a split dose over 3 days; Etoposide 100 mg/day for 3 days; Vindesine 3 mg/m^2^ for 1 day; Dexamethasone 10 mg/day for 7 days); G-CSF 10 μg/kg/day s.c. as a split dose twice daily for 5-7 days. |
|  | MEOD + MTX + G-CSF SD | MEOD; MTX 2 g/m^2^ for 1 day; G-CSF 10 μg/kg/day s.c. as a split dose twice daily for 5-7 days. |
| Zhu 2008 [65] | Chemotherapy + G-CSF RD | Disease-based mobilizing chemotherapy (CY, teniposide, vincristine and prednisone for NHL; mitoxantrone, cytarabine and teniposide for AML); G-CSF 5 μg/kg/day s.c. for median of 7.5 days. |
|  | Chemotherapy + G-CSF RD + IL-11 | Disease-based mobilizing chemotherapy; G-CSF 5 μg/kg/day s.c. for median of 5.5 days; rhIL-11 50 μg/kg/day s.c. for median of 4 days. |
| Zhu 2018 [66] | G-CSF SD + Plerixafor SD | G-CSF 10 μg/kg/day on days 1-8; Plerixafor 0.24 mg/kg/day s.c. in the evening on days 4-7. |
|  | G-CSF SD | G-CSF 10 μg/kg/day on days 1-8. |
| Zhu 2019 [67] | CE + G-CSF RD + TPO | CE: CY 2 g/m^2^ i.v. on days 1-2, VP-16 200 mg/m^2^ i.v. on day 1; G-CSF (Filgrastim) 7.5 μg/kg/day, rhTPO 15,000 U/day s.c. from day 4 to the end of collection. |
|  | CE + G-CSF RD | CE; G-CSF (Filgrastim) 7.5 μg/kg/day s.c. from day 4 to the end of collection. |

Abbreviation: AML, acute myelocytic leukemia; CE, cyclophosphamide, etoposide; CEP, cyclophosphamide, etoposide, and cisplatin; CMD, cyclophosphamide, mitoxantrone and dexamethasone; CY, cyclophosphamide; DHAP, dexamethasone, high-dose cytarabine, and cisplatin; ESHAP, etoposide, methylprednisolone, high-dose cytarabine, and cisplatin; EPO, erythropoietin; FD, fixed dose; GM-CSF, granulocyte-macrophage colony-stimulating factor; G-CSF: granulocyte colony-stimulating factor; ICE, ifosfamide, carboplatin, etoposide; ID-AraC, intermediate-dose cytarabine; IEV, ifosfamide, etoposide and epirubicin; IL-11, interleukin 11; i.v. intravenously inject; MA, methotrexate and cytosine arabinoside; MEOD, mitoxantrone, etoposide, vindesine and dexamethasone; MM, multiple myeloma; MTX, methotrexate; NHL, non-Hodgkin lymphoma; RD, reduced dose; s.c., [subcutaneous](javascript:;)ly inject; SCF, stem cell factor; SD, standard dose; TPO, thrombopoietin; VP-16, etoposide; YF-H-2015005, a new CXCR4 antagonist.
