# Supplementary material for: Efficacy of hematopoietic stem cell mobilization regimens in patients with hematological malignancies: a systematic review and network meta-analysis of randomized controlled trials
Source: Stem Cell Res Ther. 2022 Mar 22;13:123. doi: 10.1186/s13287-022-02802-6 (PMC8939102; doi:10.1186/s13287-022-02802-6)
Supplement: Supplementary file 3 — Additional file 3: Table S3. The characteristics and mobilization results of the 44 trials included in review. [file 13287_2022_2802_MOESM3_ESM.docx]

**Supplementary Table 3. The characteristics and mobilization results of the 44 trials included in review.**

| Study | Minimal mobilization target | Optimal mobilization target | Maximum No. of apheresis | Mobilization regimen | No. of patients | Total CD34^+^ cells (× 10^6^/kg) collected**^*^** | Patients reaching minimal target (%) | Patients reaching optimal target (%) | Days to neutrophil engraftment**^#^** | Days to platelet engraftment^#^ |
| --- | --- | --- | --- | --- | --- | --- | --- | --- | --- | --- |
| Arora 2004 [24] | ≥ 2 × 10^6^/kg | ≥ 5 × 10^6^/kg | NA | CMD + G-CSF RD | MM: 35 | 16.4 (4.0-37.0) | 100 | 91.4 | NA | NA |
|  |  |  |  | CMD + GM-CSF RD | MM: 37 | 12.8 (9.9-18.8) | 100 | 89.2 | NA | NA |
| Bhamidipati 2017 [25] | ≥ 2 × 10^6^/kg | ≥ 5 × 10^6^/kg | 4 | Biosimilar G-CSF SD + Plerixafor SD | MM: 43; NHL: 3 | NA | 100 | 96 | 11 | 18 |
|  |  |  |  | G-CSF SD + Plerixafor SD | MM: 43; NHL: 8 | NA | 100 | 96 | 11 | 18 |
| Bouko 2013 [26] | ≥ 2 × 10^6^/kg | ≥ 4 × 10^6^/kg | 4 | G-CSF SD | MM: 23 | 8.11 (2.09-17.07) | 100 | 82 | NA | NA |
|  |  |  |  | Pegfilgrastim 12 mg | MM: 23 | 7.05 (2.16-15.57) | 100 | 81 | NA | NA |
|  |  |  |  | Pegfilgrastim 18 mg | MM: 22 | 7.21 (1.65-13.1) | 98.5 | 90.5 | NA | NA |
| Bourin 2004 [27] | NA | ≥ 5 × 10^6^/kg | 2 | CY + G-CSF RD | MM: 67 | 16 | NA | NA | NA | NA |
|  |  |  |  | SCF + G-CSF SD | MM: 71 | 15 | NA | NA | NA | NA |
| Chen 2010 [28] | ≥ 2 × 10^6^/kg | NA | 3 | CY + G-CSF RD | NHL: 14 | 3.04 ± 0.74 | 100 | NA | 9.1 ± 2.3 | 10.3 ± 3.5 |
|  |  |  |  | MA + G-CSF RD | NHL: 11 | 5.45 ± 4.62 | 100 | NA | 8.5 ± 4.3 | 9.2 ± 2.9 |
| Copelan 2009 [29] | ≥ 2 × 10^6^/kg | ≥ 7 × 10^6^/kg | 10 | VP-16 + G-CSF SD | NHL: 27 | 5.60 (0.15-36.13) (9.09 ± 9.77) | 85.2 | NA | 10 (9-12) | 17 (10-34) |
|  |  |  |  | Rituximab + VP-16 + G-CSF SD | NHL: 28 | 9.94 (1.40-100.07) (17.36 ± 21.89)**†** | 96.4 | NA | 10 (9-12) | 16 (10-20) |
| Czerw 2019 [30] | NA | ≥ 5 × 10^6^/kg | 3 | G-CSF SD | MM: 46 | 5.9 (0-11) | NA | 70 | 11 (9-12) | 17 (14-21) |
|  |  |  |  | ID-AraC + G-CSF SD | MM: 44 | 20.2 (2.9-59.4)**†** | NA | 98**†** | 10 (8-11)**†** | 15 (13-19)**†** |
| Demuynck 1995 [31] | NA | NA | NA | CY + G-CSF SD | MM: 11 | 12.66 (2.19-52.58) | NA | NA | NA | NA |
|  |  |  |  | CY + GM-CSF SD | MM: 11 | 23.65 (2.03-59.36) | NA | NA | NA | NA |
| DiPersio 2009-3101 [32] | ≥ 2 × 10^6^/kg | ≥ 5 × 10^6^/kg | 4 | G-CSF SD + Plerixafor SD | NHL: 150 | 5.69 (0.03-29.22) **†** | 86.7**†** | 59.3**†** | 10 | 20 |
|  |  |  |  | G-CSF SD | NHL: 148 | 1.98 (0.06-15.00) | 47.3 | 19.6 | 10 | 20 |
| DiPersio 2009-3102 [33] | ≥ 2 × 10^6^/kg | ≥ 6 × 10^6^/kg | 4 | G-CSF SD + Plerixafor SD | MM: 148 | 10.96 (0.66-16.74) (5.84 ± 2.64)**†** | 95.3**†** | 75.7**†** | 11 | 18 |
|  |  |  |  | G-CSF SD | MM: 154 | 3.98 (1.76-16.88) (4.41 ± 2.22） | 88.3 | 51.3 | 11 | 18 |
| Facon 1999 [34] | ≥ 2 × 10^6^/kg | ≥ 5 × 10^6^/kg | 4 | CY + SCF + G-CSF RD | MM: 55 | 12.4 (0.0-90.4)**†** | NA | 85.4**†** | 10 | 9 |
|  |  |  |  | CY + G-CSF RD | MM: 47 | 8.2 (0.3-65.9) | NA | 76.6 | 10 | 10 |
| Gazitt 2001 [35] | ≥ 2 × 10^6^/kg CD34^+^CD45^dim^ cells | NA | 4 | CY + G-CSF SD | NHL: 13 | NA | 77 | NA | 11 | 11 |
|  |  |  |  | CY + GM-CSF RD | NHL: 10 | NA | 60 | NA | 10 | 11 |
|  |  |  |  | CY + GM-CSF RD + G-CSF SD | NHL: 12 | NA | 58 | NA | 10 | 11 |
| Hart 2009 [36] | NA | ≥ 6 × 10^6^/kg | 2 | IEV + G-CSF RD | MM: 12 | 12.6 (4.2-42.2) | NA | 83 | NA | NA |
|  |  |  |  | IEV + G-CSF RD + EPO | MM: 14 | 15.4 (6.2-63.6) | NA | 100 | NA | NA |
| Hohaus 1998 [37] | NA | NA | 6 | G-CSF RD | HL: 12 | 7.6 (median) | NA | NA | 11.5 | NA |
|  |  |  |  | GM-CSF RD | HL: 14 | 5.6 (median) | NA | NA | 13 | NA |
| Jeker 2020 [38] | ≥ 2 × 10^6^/kg | ≥ 6 × 10^6^/kg | NA | Vinorelbine + G-CSF SD | MM: 67 | 11.4 (3.7-27.9)**†** | 100 | 91**†** | 11 | 12 |
|  |  |  |  | Gemcitabine + G-CSF SD | MM: 63 | 8.7 (1.3-26.3) | 100 | 79 | 12 | 13 |
| Johnsen 2011 [39] | NA | ≥ 5 × 10^6^/kg | 5 | CY + G-CSF SD | HL: 4; NHL: 12 | 8.3 (1.4-16.1) | NA | 81 | 10 (9-13) | 11 (8-16) |
|  |  |  |  | SCF + G-CSF SD | HL: 3; NHL: 13 | 5.4 (1.9-8.5) | NA | 56 | 11 (10-12) | 11 (8-16) |
| Karanth 2004 [40] | ≥ 2 × 10^6^/kg | NA | 3 | CY + G-CSF RD | MM: 18; NHL: 8; HL: 3; CLL:10 | 2.2 (0-10) | 54.6 | NA | 14 (12-21) | 14 (9-36) |
|  |  |  |  | G-CSF SD | MM: 17; NHL: 8; HL: 3; CLL:12 | 2.3 (0.1-5.8) | 70 | NA | 14 (11-39) | 15 (11-121) |
| Kim 2005 [41] | ≥ 2 × 10^6^/kg | ≥ 5 × 10^6^/kg | 6 | CY or ESHAP ± Rituximab + G-CSF SD (single dose) | MM: 13; NHL: 7 | 19.4 (4.1-52.6) | 100 | 90 | 10 | 12 |
|  |  |  |  | CY or ESHAP ± Rituximab + G-CSF SD (split dose) | MM: 12; NHL: 8 | 15.8 (5.2-70.6) | 100 | 100 | 10 | 11 |
| Kuan 2015 [42] | ≥ 2 × 10^6^/kg | NA | 3 | CY + G-CSF RD | MM: 8; Lymphoma: 27; Acute leukemia: 9 | 2.8 (IQR 5.5) | 63.6 | NA | 10 (8-12) | 9.5 (6-11) |
|  |  |  |  | CY + Pegfilgrastim 6 mg | MM: 8; Lymphoma: 28; Acute leukemia: 6 | 1.8 (IQR 4.3) | 47.6 | NA | 10 (9-11) | 9 (7-20) |
|  |  |  |  | CY + late Pegfilgrastim 6 mg | MM: 13; Lymphoma: 29; Acute leukemia: 6 | 3.1 (IQR 3.7) | 70.8 | NA | 11 (8-11) | 9 (7-13) |
| Kuruvilla 2018 [43] | ≥ 2 × 10^6^/kg | ≥ 5 × 10^6^/kg | 4 | G-CSF SD + Plerixafor FD | NHL: 30 | 5.35 (median) | 93.3 | 60 | NA | NA |
|  |  |  |  | G-CSF SD + Plerixafor SD | NHL: 31 | 5.24 (median) | 90.3 | 54.8 | NA | NA |
| Liu 2021 [44] | ≥ 2 × 10^6^/kg | ≥ 5 × 10^6^/kg | 4 | G-CSF SD + YF-H-2015005 | NHL: 51 | 5.10 (0.27-11.12) **†** | 86.3**†** | 56.9**†** | 11 | 13 |
|  |  |  |  | G-CSF SD | NHL: 50 | 1.67 (0.17-8.09) | 38 | 12 | 11 | 13 |
| Lonial 2004 [45] | ≥ 2 × 10^6^/kg | ≥ 5 × 10^6^/kg | NA | Chemotherapy + G-CSF SD | MM: 13; Lymphoma: 5 | 7.0 ± 5.4 | NA | NA | NA | NA |
|  |  |  |  | Chemotherapy + G-CSF RD + GM-CSF RD | MM: 10; Lymphoma: 7 | 14.3 ± 5.9 | NA | NA | NA | NA |
| Manko 2014 [46] | ≥ 2 × 10^6^/kg | ≥ 5 × 10^6^/kg | 3 | Chemotherapy + Biosimilar G-CSF SD | MM: 25; NHL: 14; HL: 11; AML: 4 | 9.1 (0-23) | NA | NA | NA | NA |
|  |  |  |  | Chemotherapy + G-CSF SD | MM: 21; NHL: 13; HL: 15; AML: 2; ALL: 1 | 9.4 (6-48) | NA | NA | NA | NA |
| Marchesi 2018 [47] | NA | ≥ 4 × 10^6^/kg | NA | Chemotherapy + G-CSF RD | NHL: 14; HL: 7 | 6.67 (1.65-15.3) | NA | 90.4 | 12 | 14 |
|  |  |  |  | Chemotherapy + Biosimilar G-CSF SD | NHL: 18; HL: 3 | 6.61 (3.46-18.5) | NA | 90.4 | 12 | 14 |
| Matsue 2018 [48] | ≥ 2 × 10^6^/kg | ≥ 5 × 10^6^/kg | 4 | G-CSF SD + Plerixafor SD | NHL: 16 | 5.45 ± 2.55**†** | 93.8 | 56.3**†** | NA | NA |
|  |  |  |  | G-CSF SD | NHL: 16 | 2.09 ± 1.69 | 31.3 | 6.3 | NA | NA |
| Milone 2003 [49] | NA | ≥ 5 × 10^6^/kg | NA | G-CSF SD | NHL: 14; HL: 12 | 5.4 (1.5-15) | NA | 50 | 11.2 (9-15) | 12.2 (10-14) |
|  |  |  |  | CY + G-CSF SD | NHL: 19; HL: 7 | 6.8 (2.7-24) | NA | 61 | 11.8 (10-16) | 14.5 (11-23) |
| Nahi 2019 [50] | ≥ 2 × 10^6^/kg | ≥ 6 × 10^6^/kg | 4 | G-CSF SD | MM: 10 | 6.8 ± 2.9 | 100 | 40 | NA | NA |
|  |  |  |  | G-CSF SD + Plerixafor SD | MM: 10 | 8.5 ± 3.3 | 100 | 70 | NA | NA |
| Narayanasami 2001 [51] | NA | NA | 3 | G-CSF SD | NHL: 15; HL: 8 | 2.5 (0.3-12.4) | NA | NA | 11 | 14 |
|  |  |  |  | CY + G-CSF SD | NHL: 17; HL: 7 | 7.2 (0.3-44.8)**†** | NA | NA | 11 | 13 |
| Orciuolo 2011 [52] | NA | ≥ 6 × 10^6^/kg | NA | CY + G-CSF (Lenograstim) SD | MM: 72 | NA | NA | 73.6 | NA | NA |
|  |  |  |  | CY + G-CSF (Filgrastim) SD | MM: 76 | NA | NA | 68.4 | NA | NA |
| Ozcelik 2009 [53] | ≥ 2 × 10^6^/kg | ≥ 4 × 10^6^/kg | 3 | CE + early G-CSF SD | MM: 11; Lymphoma: 14 | 10.54 (0.11-37.27) | 84 | NA | NA | NA |
|  |  |  |  | CE + late G-CSF SD | MM: 15; Lymphoma: 8 | 10.81 (0.17-49.83) | 87 | NA | NA | NA |
| Pavone 2002 [54] | ≥ 2 × 10^6^/kg | NA | 3 | DHAP + G-CSF RD | NHL: 38 | 5.9 (mean) | 85.3 | NA | 9 | 13 |
|  |  |  |  | CY + G-CSF RD | NHL: 34 | 7.06 (mean) | 89.5 | NA | 10 | 10 |
| Ri 2017 [55] | ≥ 2 × 10^6^/kg | ≥ 6 × 10^6^/kg (within 2 apheresis) | 4 | G-CSF SD + Plerixafor SD | MM: 7 | 7.55 ± 2.32**†** | 100 | 71.4**†** | NA | NA |
|  |  |  |  | G-CSF SD | MM: 7 | 3.67 ± 1.25 | 85.7 | 0 | NA | NA |
| Russell 2008 [56] | ≥ 2 × 10^6^/kg | ≥ 5 × 10^6^/kg | 5 | ICE + G-CSF RD | NHL: 32 | 4.3 (0.0-11.7) | 72 | 56 | 11 | 10.5 |
|  |  |  |  | ICE + Pegfilgrastim 6 mg | NHL: 31 | 4.9 (0.0-11.4) | 69 | 41 | 12 | 11 |
|  |  |  |  | ICE + Pegfilgrastim 12 mg | NHL: 29 | 5.1 (0.0-14.3) | 59 | 45 | 11 | 11 |
| Samaras 2018 [57] | NA | ≥ 4 × 10^6^/kg | 3 | Vinorelbine + G-CSF SD | MM: 20 | 7.65 (5.0-11.2) (median, IQR) | NA | 100 | 10 | 12 |
|  |  |  |  | Vinorelbine + G-CSF RD | MM: 40 | 6.87 (5.4-10.2) (median, IQR) | NA | 100 | 10 | 12 |
| Silvennoinen 2016 [58] | ≥ 3 × 10^6^/kg | ≥ 6 × 10^6^/kg | 4 | CY + G-CSF RD | MM: 34 | 6.7 (2.2-12.4)**†** | 94 | 62 | 14 (9-28) | 12 (8-30) |
|  |  |  |  | G-CSF SD | MM: 35 | 5.3 (2.4-12.4) | 77 | 50 | 14 (11-27) | 11 (8-30) |
| Skopec 2017 [59] | ≥ 2 × 10^6^/kg | NA | 5 | G-CSF SD | MM: 20 | 5.05 (2.71-13.74) | 95.3 | NA | 13 (12-26) | 16.5 (9-36) |
|  |  |  |  | Pegfilgrastim 12 mg | MM: 19 | 4.66 (1.91-8.37) | 90.5 | NA | 13 (12-21) | 16 (12-26) |
| Stiff 2000 [60] | NA | ≥ 5 × 10^6^/kg | 5 | SCF + G-CSF SD | NHL: 34; HL: 18 | 3.6 (median) | NA | 44.4**†** | 10 | 12 |
|  |  |  |  | G-CSF SD | NHL: 38; HL: 18 | 2.4 (median) | NA | 16.7 | 10 | 12 |
| Valtola 2016 [61] | ≥ 3 × 10^6^/kg | ≥ 6 × 10^6^/kg | NA | CY + G-CSF RD | MM: 17 | 6.7 (3.6-12.1) | NA | NA | 12 (9-21) | 12 |
|  |  |  |  | G-CSF SD | MM: 19 | 6.0 (2.8-10.7) | NA | NA | 13 (10-21) | 12 |
| Vela-Ojeda 2000 [62] | ≥ 2 × 10^6^/kg | NA | 6 | Ifosfamide + GM-CSF RD | MM: 7; NHL: 14; HL: 7 | 3.14 (0.9-11.8) | 86 | NA | 16 (10-22) | 13 (10-24) |
|  |  |  |  | CY + GM-CSF RD | MM: 7; NHL: 12; HL: 9 | 5.33 (0.08-32) | 89 | NA | 17 (11-33) | 15 (7-41) |
| Weaver 1998 [63] | ≥ 2.5 × 10^6^/kg | ≥ 5 × 10^6^/kg | 10 | CE + G-CSF RD | NHL: 31; HL: 10 | 19.77 (0.11-84.32) | 90 | 85 | 9 (8-19) | 9 (4-34) |
|  |  |  |  | CEP + G-CSF RD | NHL: 31; HL: 9 | 9.39 (0.03-42.05) | 89 | 71 | 9 (8-23) | 10 (6-28) |
| Zhang 2014 [64] | ≥ 2 × 10^6^/kg | NA | 3 | MEOD + MTX + G-CSF SD | NHL: 33 | 14.36 (2.51-66.78)**†** | 100 | NA | 8 (6-10)**†** | 12 (10-27)**†** |
|  |  |  |  | MEOD + G-CSF SD | NHL: 34 | 5.3 (0.30-24.38) | 94 | NA | 11 (10-13) | 13 (11-19) |
| Zhu 2008 [65] | NA | NA | 4 | Chemotherapy + G-CSF RD | NHL: 5; AML: 3 | 6.3 (2.9-19.0) | NA | NA | 13 (10-15) | 13 (11-21) |
|  |  |  |  | Chemotherapy + G-CSF RD + IL-11 | NHL: 7; AML: 1 | 5.6 (4.5-7.3) | NA | NA | 10.5 (9-13)**†** | 11.5 (9-13)**†** |
| Zhu 2018 [66] | ≥ 2 × 10^6^/kg | ≥ 5 × 10^6^/kg | 4 | G-CSF SD + Plerixafor SD | NHL: 50 | NA | 88**†** | 62**†** | 10 (9-10) | 19 (17-21) |
|  |  |  |  | G-CSF SD | NHL: 50 | NA | 66 | 20 | 10 (10-11) | 17 (16-19) |
| Zhu 2019 [67] | ≥ 2 × 10^6^/kg | ≥ 5 × 10^6^/kg | 3 | CE + G-CSF RD + TPO | NHL: 40 | 6.35 (2.3-22.7)**†** | 100**†** | 72.9**†** | 12 (8-15) | 12 (11-17） |
|  |  |  |  | CE + G-CSF RD | NHL: 38 | 3.3 (0.5-16.2) | 86 | 41.3 | 12 (8-14) | 14 (10-16) |

**^*^**: Data are presented as median and range or/and mean ± standard deviation.

^#^: Days to neutrophil and platelet engraftment after transplantation are presented as median with or without range or mean ± standard deviation.

**†**: Results with significant diffrences compared with control regimens (*P* < 0.05).

Abbreviation: AML, acute myelocytic leukemia; CE, cyclophosphamide, etoposide; CEP, cyclophosphamide, etoposide, and cisplatin; CMD, cyclophosphamide, mitoxantrone and dexamethasone; CLL, chronic lymphocytic leukemia; CY, cyclophosphamide; DHAP, dexamethasone, high-dose cytarabine, and cisplatin; ESHAP, etoposide, methylprednisolone, high-dose cytarabine, and cisplatin; EPO, erythropoietin; FD, fixed dose; G-CSF: granulocyte colony-stimulating factor; GM-CSF, granulocyte-macrophage colony-stimulating factor; HL, Hodgkin lympoma; ICE, ifosfamide, carboplatin, etoposide; ID-Arac, intermediate-dose cytarabine; IEV, ifosfamide, etoposide and epirubicin; IL-11, interleukin 11; MA, methotrexate and cytosine arabinoside; MEOD, mitoxantrone, etoposide, vindesine and dexamethasone; MM, multiple myeloma; MTX, methotrexate; NHL, non-Hodgkin lymphoma; NA, not available; RD, reduced dose; SCF, stem cell factor; SD, standard dose; TPO, thrombopoietin; VP-16, etoposide; YF-H-2015005, a new CXCR4 antagonist.
