# Supplementary material for: Efficacy of hematopoietic stem cell mobilization regimens in patients with hematological malignancies: a systematic review and network meta-analysis of randomized controlled trials
Source: Stem Cell Res Ther. 2022 Mar 22;13:123. doi: 10.1186/s13287-022-02802-6 (PMC8939102; doi:10.1186/s13287-022-02802-6)
Supplement: Supplementary file 4 — Additional file 4: Table S4. Results of risk of bias assessment. [file 13287_2022_2802_MOESM4_ESM.docx]

**Supplementary Table 4. Results of risk of bias assessment.**

| **Study** | **Random sequence generation** | **Allocation concealment** | **Blinding of participants and personnel** | **Blinding of outcome assessment** | **Incomplete outcome data** | **Selective reporting** | **Other bias** |
| --- | --- | --- | --- | --- | --- | --- | --- |
| ***Studies for MM*** |  |  |  |  |  |  |  |
| Bouko 2013 [26] | Unclear | Unclear | Unclear | Low | Low | Low | Low |
| Czerw 2019 [30] | Unclear | Unclear | High | Low | Low | Low | Low |
| DiPersio 2009-3102 [32] | Low | Unclear | Low | Low | Low | Low | Low |
| Nahi 2019 [50] | Low | Low | High | Low | Low | Low | Low |
| Ri 2017 [55] | Low | Low | High | Low | Low | Low | Low |
| Silvennoinen 2016 [58] | Low | Unclear | Unclear | Low | Low | Low | Low |
| Skopec 2017 [59] | Low | Unclear | Unclear | Low | Low | Low | Low |
| Valtola 2016 [61] | Unclear | Unclear | Unclear | Low | Low | Low | Low |
| ***Studies for NHL*** |  |  |  |  |  |  |  |
| DiPersio 2009-3101 [33] | Unclear | Unclear | Low | Low | Low | Low | Low |
| Kuruvilla 2018 [43] | Low | Low | High | Low | Low | Low | Low |
| Liu 2021 [44] | Unclear | Unclear | Low | Low | Low | Low | Low |
| Matsue 2018 [48] | Low | Low | High | Low | Low | Low | Low |
| Zhu 2018 [66] | Low | Low | Low | Low | Low | Low | Low |
