# Supplementary material for: Efficacy of hematopoietic stem cell mobilization regimens in patients with hematological malignancies: a systematic review and network meta-analysis of randomized controlled trials
Source: Stem Cell Res Ther. 2022 Mar 22;13:123. doi: 10.1186/s13287-022-02802-6 (PMC8939102; doi:10.1186/s13287-022-02802-6)
Supplement: Supplementary file 5 — Additional file 5: Table S5. Results of meta-analysis regarding the rate of reaching optimal target. [file 13287_2022_2802_MOESM5_ESM.docx]

**Supplementary Table 5. Results of meta-analysis regarding the rate of reaching optimal target.**

| **Mobilization regimens** | **Mean rank** | **OR (95% CrI)** | **Probability of being best** | **SUCRA** |
| --- | --- | --- | --- | --- |
| ***For MM*** |  |  |  |  |
| ID-AraC + G-CSF SD | 1.1 | **27.1 (4.23, 771)*** | 94.09% | 0.99 |
| G-CSF SD + Plerixafor SD | 2.7 | **3.03 (1.89, 4.95)*** | 0.59% | 0.66 |
| Pegfilgrastim 18 mg | 3.4 | 2.30 (0.36, 20.8) | 4.44% | 0.52 |
| CY + G-CSF RD | 3.9 | 1.66 (0.46, 6.33) | 0.61% | 0.41 |
| Pegfilgrastim 12 mg | 4.8 | 1.00 (0.20, 5.07) | 0.27% | 0.23 |
| G-CSF SD | 5.1 | -- | 0 | 0.18 |
| ***For NHL*** |  |  |  |  |
| G-CSF SD + YF-H-2015005 | 1.6 | **10.3 (3.86, 30.9)*** | 57.64% | 0.80 |
| G-CSF SD + Plerixafor FD | 2.0 | **8.24 (2.67, 25.9)*** | 35.16% | 0.68 |
| G-CSF SD + Plerixafor SD | 2.4 | **6.59 (5.27, 10.4)*** | 7.20% | 0.52 |
| G-CSF SD | 4.0 | -- | 0 | 0 |
