# Supplementary figures and images for: Efficacy of hematopoietic stem cell mobilization regimens in patients with hematological malignancies: a systematic review and network meta-analysis of randomized controlled trials
Source: Stem Cell Res Ther. 2022 Mar 22;13:123. doi: 10.1186/s13287-022-02802-6 (PMC8939102; doi:10.1186/s13287-022-02802-6)

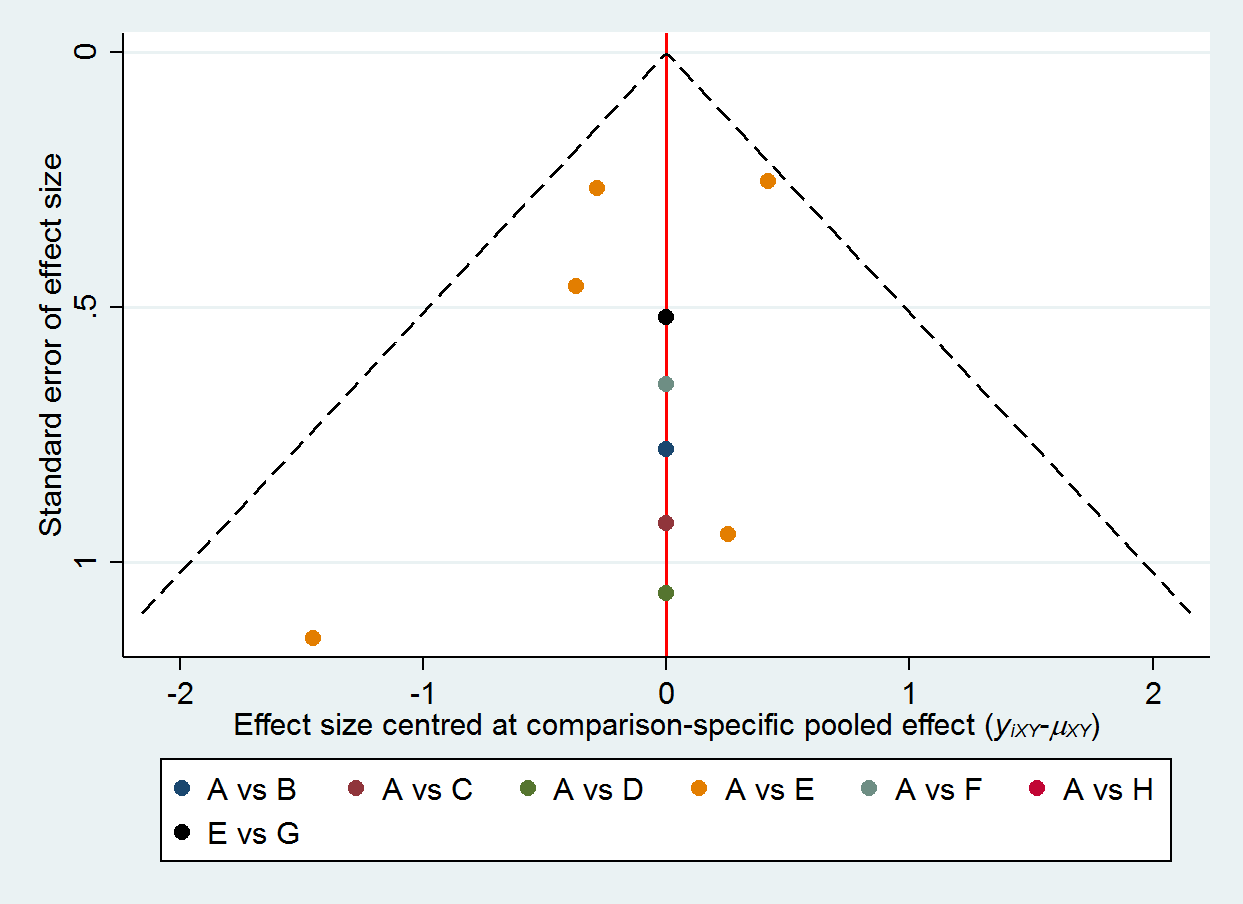

Supplement: Supplementary file 6 — Additional file 6: Figure S1. The comparison-adjusted funnel plot. A, G-CSF SD; B, Pegfilgrastim 12 mg; C, Pegfilgrastim 18 mg; D, ID-AraC + G-CSF SD; E, G-CSF SD + Plerixafor SD; F, CY + G-CSF RD; G, G-CSF SD + Plerixafor FD; H, G-CSF SD + YF-H-2015005. [file 13287_2022_2802_MOESM6_ESM.tif]

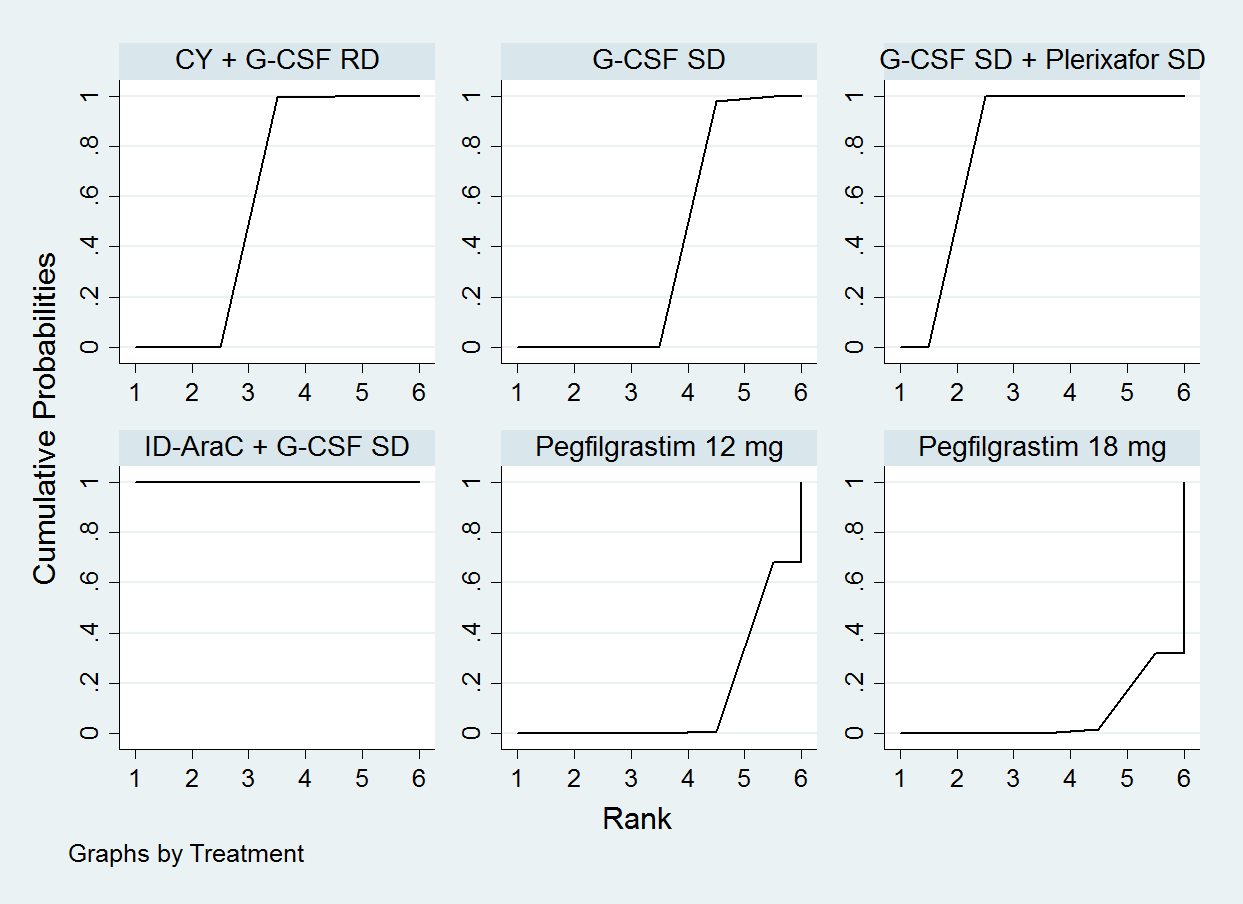

Supplement: Supplementary file 7 — Additional file 7: Figure S2. The SUCRA graphs regarding the number of total CD34+ cells collected for patients with MM. [file 13287_2022_2802_MOESM7_ESM.tif]

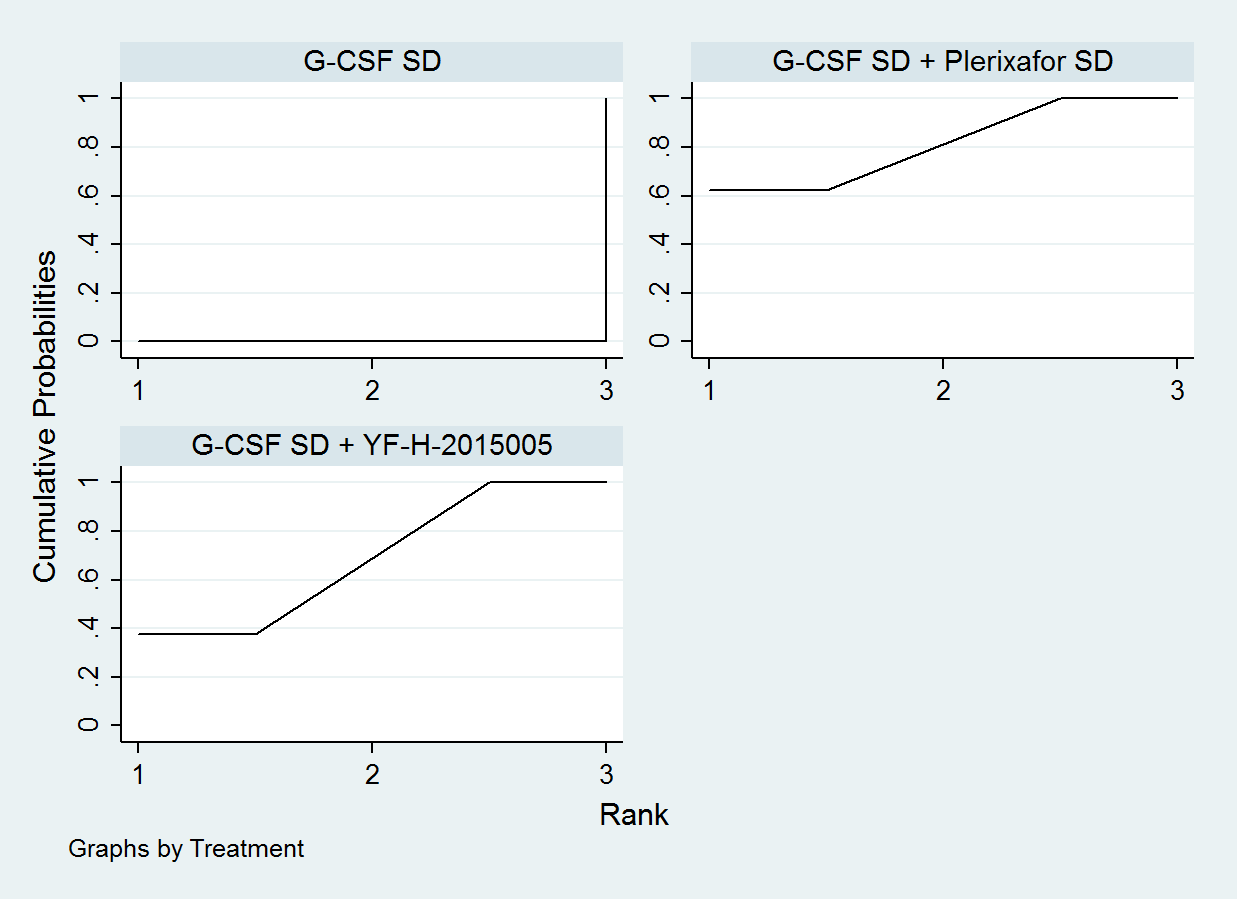

Supplement: Supplementary file 8 — Additional file 8: Figure S3. The SUCRA graphs regarding the number of total CD34+ cells collected for patients with NHL. [file 13287_2022_2802_MOESM8_ESM.tif]

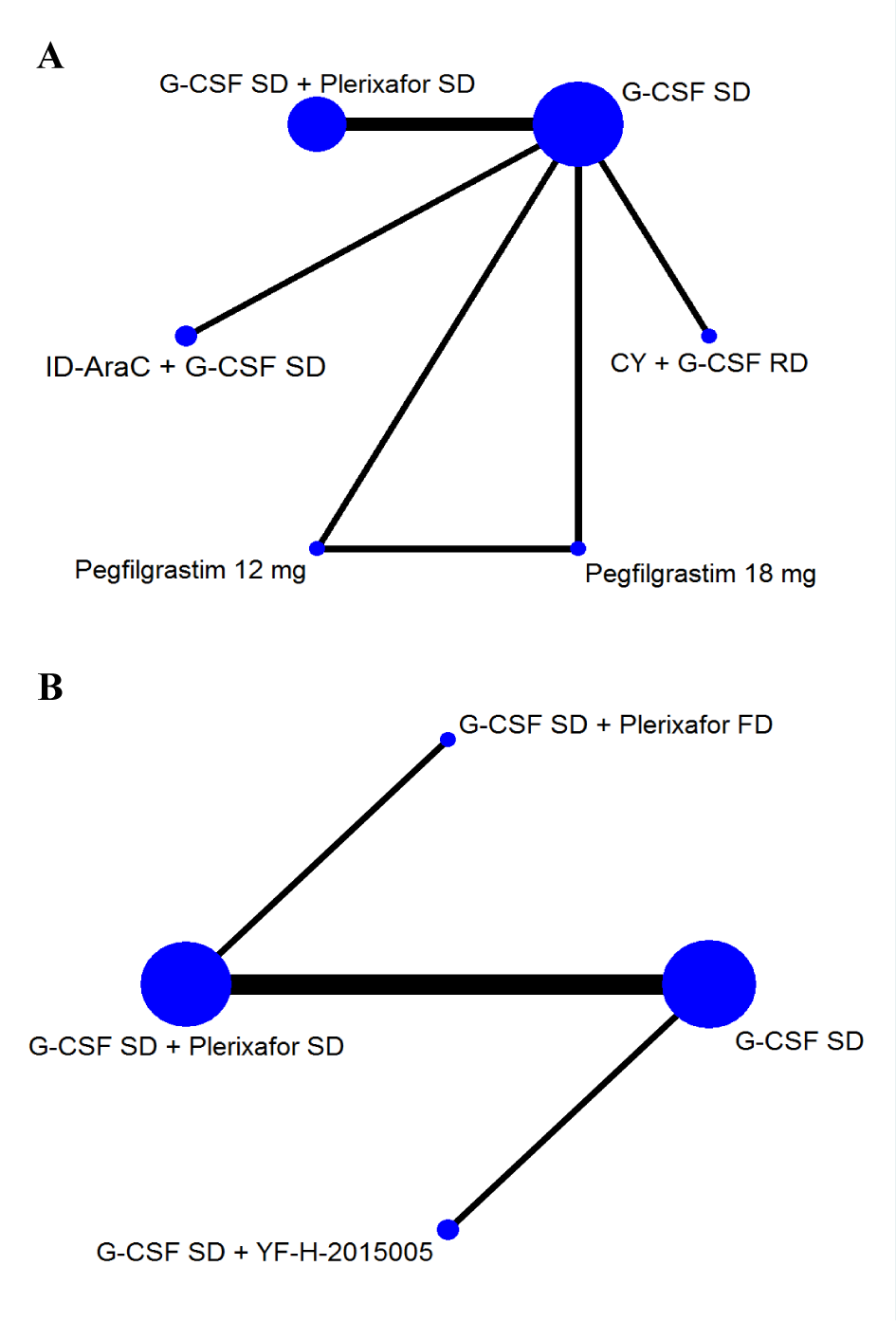

Supplement: Supplementary file 9 — Additional file 9: Figure S4. Network plot depicting all direct comparisons in included trials with data about the successful rate of reaching optimal target for patients with MM (A) and NHL (B). [file 13287_2022_2802_MOESM9_ESM.tif]

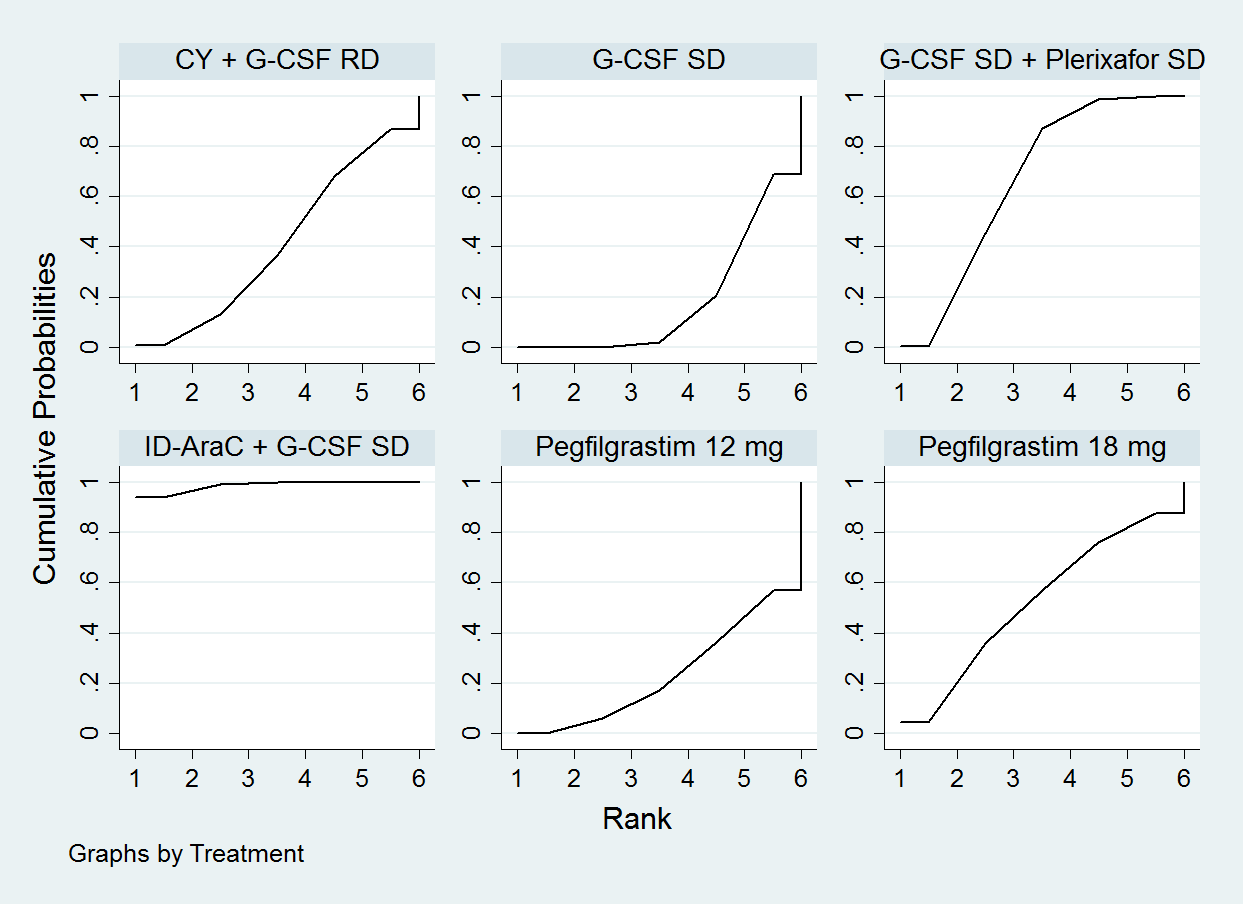

Supplement: Supplementary file 10 — Additional file 10: Figure S5. The SUCRA graphs regarding the successful rate of reaching optimal target for patients with MM. [file 13287_2022_2802_MOESM10_ESM.tif]

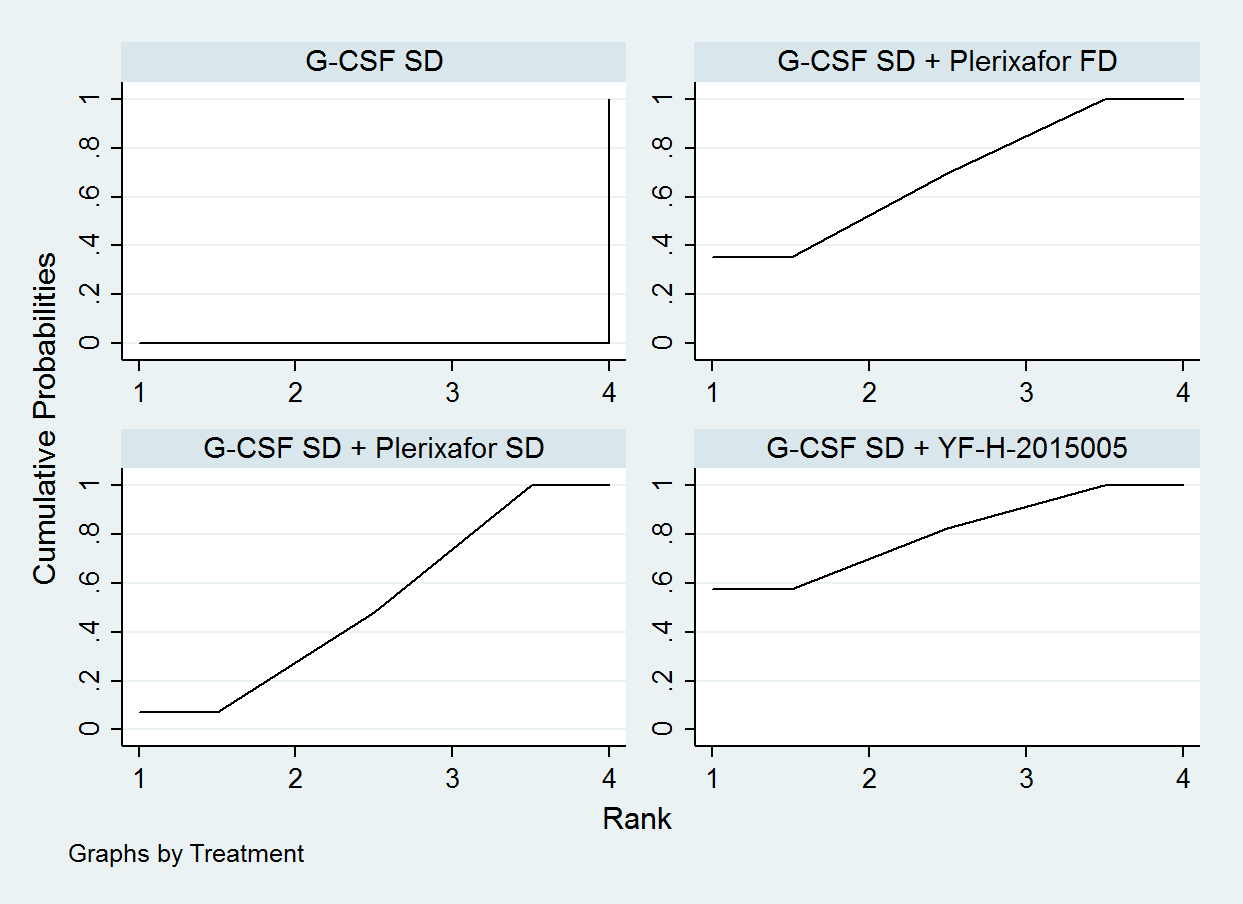

Supplement: Supplementary file 11 — Additional file 11: Figure S6. The SUCRA graphs regarding the successful rate of reaching optimal target for patients with NHL. [file 13287_2022_2802_MOESM11_ESM.tif]
